# Patient Centred Medical Home (PCMH) transitions in western Sydney, Australia: a qualitative study

**DOI:** 10.1186/s12913-020-05123-7

**Published:** 2020-04-06

**Authors:** Christine Metusela, Tim Usherwood, Kenny Lawson, Lisa Angus, Walter Kmet, Shahana Ferdousi, Jennifer Reath

**Affiliations:** 1 Department of General Practice, School of Medicine, Western Sydney University, Building 30.3.24 Campbelltown Campus, Locked Bag 1797, Penrith, NSW 2751 Australia; 2 General Practice, School of Medicine, University of Sydney, Sydney, Australia; 3 Translational Health Research Institute, Western Sydney University, Sydney, Australia; 4 Providence Center for Outcomes Research & Education, Portland, OR USA; 5 Macquarie University Hospital and Clinical Services, Sydney, Australia; 6 WentWest Ltd, Western Sydney Primary Health Network, Blacktown, Australia

**Keywords:** Primary health care, Primary care, General practice, Patient Centred medical home, Continuity of patient care, Qualitative research

## Abstract

**Background:**

Patient Centred Medical Homes (PCMHs), increasingly evidenced to provide high quality primary care, are new to Australia. To learn how this promising new healthcare model works in an Australian setting we explored experiences of healthcare providers in outer urban Sydney, where a number of practices are transitioning from traditional Australian general practice models to incorporate elements of PCMH approaches.

**Methods:**

We collected qualitative data from semi-structured interviews with healthcare providers working in a range of transitioning practices and thematically analysed the data. We interviewed 35 participants including general practitioners, practice managers and practice nurses from 25 purposively sampled general practices in western Sydney, Australia, seeking maximal variation in practice size, patient demographics and type of engagement in practice transformation.

**Results:**

Interviewees described PCMH transformation highlighting the importance of whole of practice engagement with a shared vision; key strategies for transformation to PCMH models of care including leadership, training and supportive information technology; structures and processes required to provide team-based, data-driven care; and constraints such as lack of space and the current Australian fee-for-service general practice funding model. They also reported their perceptions of early outcomes of the PCMH model of care, describing enhanced patient and staff satisfaction and also noting fewer hospital admissions, as likely to reduce costs of care.

**Conclusions:**

Our study exploring the experience of early adopters of PCMH models of care in Australia, informs the international movement towards PCMH models of care. Our findings provide guidance for practices considering similar transitions and describe the challenges of such transitions within a fee-for-service payment system.

## Background

Health systems with high-performing primary healthcare have been shown to provide improved and more equitable health outcomes at lower cost [1]. The transformation of primary care practices into Patient Centred Medical Homes (PCMHs) has been proposed as a means of enabling high quality healthcare [2, 3]. In the United States the PCMH model has been active and evolving for over a decade, with the American Academy of Family Physicians launching the first large-scale national demonstration of the PCMH in June 2006 [4]. The PCMH model was proposed as a means of improving primary care through provision of team-based, patient-centred care for a registered patient cohort, thereby enhancing patient experience, and improving quality of care [5, 6]. The model is based on key attributes and functions that include patient-centredness, comprehensive and coordinated care, accessible services, and a commitment to quality and safety [7]. More recently PCMH practice transformation has been described based on 10 “building blocks” characterising high-performing primary care [8]. These “building blocks” consist of four foundation blocks: engaged leadership, data-driven improvement, empanelment (linking the patient to a care team and a primary care physician), and team-based care and six higher order blocks: patient-team partnership, population management, continuity of care, prompt access to care, comprehensiveness and care coordination, and template of the future [8].

Outcomes reported as potentially associated with PCMH transformation include improved patient experience of care [2], enhanced work life of healthcare providers [9], better population health outcomes [2, 3], reduced hospitalisations and costs to individuals and the health system [2, 3, 10]. These outcomes align with the Quadruple Aim [11] of improving patient and healthcare provider experience, and the health of the population while reducing costs. Positive outcomes in primary care reorganisation have been highlighted in the United States [12], as well as in Canada [13] and New Zealand [14]. However, some studies report mixed results in terms of cost savings and quality of healthcare [3]. Barriers and challenges to implementing a PCMH model have also been reported. The time and resources required to implement the model and to transform work processes incur substantial costs, both one-time and ongoing [5, 15, 16]. Studies that examine patient experiences of PCMH have been mainly limited to patient-provider relationship and access to care, whereas other aspects such as patient engagement, activation, shared decision-making and patient experience with other practice staff have received less investigation [17].

Australia, as many other countries, struggles with an ageing population, increasing prevalence of chronic disease and burgeoning healthcare costs [18–20]. Hence there is a pressing need for high-performing healthcare with a focus on preventative health and team management of chronic disease [19, 21]. The Royal Australian College of General Practitioners (RACGP) has promoted the PCMH model as a means of enhancing quality of primary care [20, 22]. This need for high performing primary care is clearly apparent in western Sydney, where the population of 1.9 million includes some living in areas of considerable disadvantage, up to 44% born overseas, and one of the largest Aboriginal and Torres Strait Islander populations in Australia [23–25].

Primary healthcare services in Australia are delivered in settings such as general practices, community health centres, Aboriginal community controlled health services, and allied health practices [26]. General practice is usually the first point of contact for patients accessing healthcare services [27]. A universal health insurance scheme (Medicare) funds healthcare provided in general practice, although approximately 14% [18, 28] of general practitioners (GPs) charge a higher rate than is reimbursed under this scheme thus leaving patients out of pocket. There are some payments available to GPs for demonstrating quality healthcare. At the time of our study, these included addressing quality indicators in areas such as diabetes management, cervical screening, provision of afterhours care and engagement in teaching. There is no component of general practice funding in Australia for patient registration with a particular practice, and patients often consult with more than one GP, sometimes in different practices [27]. Approximately 95% of general practice income is derived from fee-for-service Medicare payments to GPs for episodic care [29]. Practices engaging in high rates of short consultations are better remunerated than those providing fewer, longer consultations [30].

Transitioning to a PCMH model entails the practice adopting a team-based approach to a more patient centred model of care, where metrics are established and monitored to improve care quality, where care is co-ordinated including beyond the practice, and there is enhanced access to services and improved communication with patients. There is a focus on building a multidisciplinary team that includes allied health professionals as well as a shift to being more nurse-driven. In this model patients are also encouraged to be involved in their own healthcare [2, 5, 6].

WentWest Ltd., operates as the Western Sydney Primary Health Network (PHN), an independent organisation contracted to the Australian Government and tasked with increasing the efficiency and effectiveness of primary healthcare, particularly for those patients at risk of poor health outcomes [31]. There are approximately 350 general practices in western Sydney, ranging from solo practitioners to large group practices, including both private and corporately managed services. The PCMH model is viewed by WentWest as a key means of improving primary care and the WentWest Strategic Plan (2016–2019) has a focus on supporting practices to implement PCMH models of care [32]. This support has included practice-based training, data extraction and analysis, assistance with quality improvement, and other resources from WentWest. Since 2014, 15 practices have been engaged in transformation to a PCMH model of care. Eight continue to transition, supported by WentWest.

In parallel with the PCMH transitions described in this research, two other programs aimed at enhancing primary care in western Sydney are being implemented. Of the 350 general practices in western Sydney, approximately 60 (including the current eight PCMH transitioning practices) are involved in the Western Sydney Integrated Care Program (WSICP) [33] originally funded as a demonstration project by the New South Wales Government. The WSICP was designed to provide co-ordinated and integrated care aimed at improving management of patients with three chronic diseases (diabetes, cardiac failure and chronic obstructive pulmonary disease) likely to result in attendance and or admission to hospital [34, 35]. Though the focus of WSICP is on the integration of healthcare across community and hospital settings, participating Integrated Care general practices have implemented a number of the “building blocks” of PCMH models of care [8, 35].

Twenty two practices, including the PCMH transitioning practices and some WSICP practices, are currently participating in the Health Care Homes (HCH) trial funded by the Australian government [36], from October 2017 until June 2021. This program was anticipated, but had not commenced at the time of our interviews. This initiative supports general practices to enrol patients with chronic and complex conditions to a practice-based program of coordinated, integrated care, tailored to their health needs. Each enrolled patient is allocated one of three payment levels based on the complexity of their health needs, with patients on the highest tier allocated the maximum payment. The HCH model uses a bundled payment model, with practices receiving a one-off establishment grant of $11,000 and monthly bundled payments that are linked to the payment tier of enrolled patients [37].

**Fig. 1:**
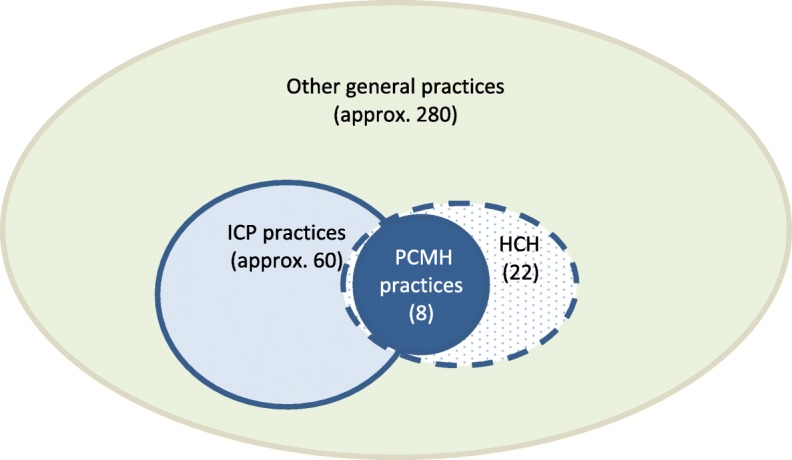
Practice types in western Sydney at the time of the study

There also remain many practices in western Sydney that are not known to be currently engaged in any large-scale practice transformation. This variety is depicted in Fig. 1.

As PCMH models are relatively new to Australia but are seen by some as the way of the future [20], it is important to understand the change process, including facilitators and barriers to implementation of this healthcare model in an Australian context. Providing evidence about performance of the PCMH model in different environments is also likely to benefit those working on improving the quality of primary care in other countries.

The aim of our research was to explore how PCMH transitions were perceived and experienced by healthcare providers in western Sydney, Australia. The different models and range of engagement with PCMH approaches in western Sydney provided a unique opportunity to explore a diversity of experience in the same geographic area. This paper presents the qualitative findings of a larger program of research that includes cost estimates of PCMH transformation [16].

## Methods

### Participants and data collection

A range of participants were recruited for interviews, including practice principals, GP contractors, practice nurses and practice managers. We purposively sampled a variety of practices, seeking maximal variation in practice size, patient demographics, and type of engagement in practice transformation. By including practices not participating in PCMH transition at the time of our research, we were able to examine their understanding of the PCMH model, and perceived facilitators and barriers to PCMH change. Former PCMH practices were included to investigate why they ceased the transition process. Integrated Care Practices and non-PCMH practices were selected to provide socio-economic variation using the Australian Bureau of Statistics Socio-Economic Indexes for Areas (SEIFA) for ranking areas according to socio-economic advantage and disadvantage [38]. Table 1 describes the decile ranking of participating practices with 10 being the most advantaged and 1 the least. Amongst the non-PCMH transitioning practices, we recruited practices across a range of likely capacity to transition to a PCMH model, based on E-health readiness, general practice accreditation status, and professional diversity of workforce. We did not select practices on the basis of HCH involvement as this program had not commenced at the time of our study.

**Table 1:** Participant sample

Practice type	Number of practice types in region	Number of participating practices	Number of individual participants	Practice size^**1**^ and SEIFA^2^ rank within Australia
Active PCMH practices	8	8 practices	8 practice principals (PP) 3 GP contractors (GPC) 4 practice managers (PM) 2 practice nurses (PN) 1 allied health practitioner (AH)	2 small; SEIFA 6;6 6 large; SEIFA 6;8;8;10;10;10
Former PCMH practices	7	2 practices	1 practice principal (PP) 1 GP contractor (GPC)	1 small; SEIFA 6 1 large; SEIFA 10
Integrated Care Program (ICP) practices	60 (approx.)	5 practices	5 practice principals (PP)	4 small; SEIFA 6;6;8;10 1 large; SEIFA 6
Non-PCMH (and Non-ICP) practices	280 (approx.)	5 high capacity (HC) practices 5 low capacity (LC) practices	9 practice principals (PP) 1 practice manager (PM)	8 small; SEIFA 6;6;6;6;6;10;10;10 2 large; SEIFA 6;6
		**25 practices**	**35 participants**	

^1^Practice size: Small (S) = ≤5 GPs; Large (L) = ≥6

^2^SEIFA decile ranking of socio-economic advantage and disadvantage, with 10 being the most advantaged and 1 being the least

To enable the collection of perspectives and experiences of PCMH transitions, a qualitative approach was used. In consultation with our Evaluation Advisory Group that provided guidance to the research team, and informed by literature, we designed semi-structured interview guides comprising open-ended questions, minimally adapted for each participant group. The interview guides are provided in Additional file 1. WentWest assisted in recruitment of participants by emailing invitations to practices and followed up this initial invitation during routine practice visits. Those willing to participate either contacted the research team directly or informed WentWest staff who contacted the research team on their behalf. Semi-structured interviews were arranged with consenting participants and conducted by CM, an experienced qualitative researcher, either face-to-face at the workplace of the participant, or by telephone according to participant preference. We continued recruitment over a period of 6 months between March and September 2017 until our sample reached 35 participants stratified across the different participant groups (Table 1), and data saturation was reached. No participants revoked consent.

The interviews were an average of 30 min in length. In accordance with best practice guidelines, interviews were recorded and transcribed verbatim. Transcripts were checked for accuracy and de-identified with participants being assigned labels. In accordance with recommended practice, transcripts were offered to participants for review [39].

### Analysis

Data were analysed thematically, using an inductive, data-driven, iterative approach, ensuring themes best reflected participant views without fitting them into a preconceived coding framework [40]. Four of the research team (CM, JR, TU, LA) individually each coded two interviews to identify categories within the data. Four transcripts were double coded and two triple coded. The research team reviewed and agreed on codes and suggested overarching themes. Three of the team further reviewed and refined the coding frame to include emerging new codes (CM, JR, TU). The revised coding frame was reviewed by the Evaluation Advisory Group to ensure validity of the analysis. NVivo 11® software was used to aid organisation of data. The complete thematic table with quotations is provided in Additional file 2. Throughout the research process, we engaged reflexively, aware that our experiences and socio-cultural backgrounds shaped our interpretation [39]. This study was approved by the Western Sydney University Human Research Ethics Committee (H12003). The COREQ checklist on qualitative reporting [41] is provided in Additional file 3.

## Results

We interviewed 35 participants from 25 general practices. Participants included practice principals, GP contractors, practice managers, practice nurses and an allied health practitioner. Eighteen participants were from the eight PCMH transitioning practices (all of which were also Integrated Care Program practices), two from former-PCMH practices, five from non-PCMH Integrated Care Program practices and 10 from non-PCMH, non-Integrated Care practices (Table 1).

We identified four overarching themes describing PCMH transformations: PCMH vision; implementation of PCMH strategies; structures and processes related to PCMH transformation; and early outcomes of a PCMH model. Table 2 lists the themes and subthemes. Each theme and related subtheme are described below and illustrated with exemplar quotations. Further quotations and analysis can be found in Additional file 2 and in the wider body of work [16].

**Table 2:** Themes and subthemes

Vision	Implementation	Structures and Processes	Outcomes
Alignment of vision	Leadership in driving change	Working together as a team	Patients: enhanced patient centred care and improved health
Engagement in realising the vision	Time required for planning and implementation	New staffing models and organisational change	Practices: improved provider satisfaction
	PCMH support and training	Staffing and space implications	Potential health system and cost efficiencies
		Data driven care utilising information technologies	
		Communication with external stakeholders	
		Challenges of fee-for-service and funding of PCMH models	

### Patient Centred Medical Home vision

A clear PCMH vision and whole of practice engagement with the vision were seen as key enabling factors for practices transitioning to a PCMH model of care. Patient Centred Medical Home transitioning practices described a “shared vision” aligned to PCMH values as crucial for transition. Similarly, participants noted that “like-mindedness” helped initiate PCMH transformation: “we came together with exactly the same philosophy” (PCMH4PP-L). Some participants described implementing PCMH aligned activities in their practice prior to joining the PCMH program:

We were already doing a lot of the things…and trying very hard to achieve some of the changes that are already part of the building blocks in terms of data management, engagement with patients, registration, having people choose their GP, trying for continuity (PCMH4PP-L).

Whole of practice engagement was also key for staff to both understand and connect with the PCMH vision and to embed PCMH values within a team-based care model. This enabled staff to “build trust between team members” (PCMH5PP-S), and to take on new roles:

…having the team engaged and making them understand why - not just what we're doing but why we're doing it and getting the buy-in is really important to making sure that they feel that their role is important. (PCMH5GPC-S)

Conversely, a lack of practice alignment with PCMH values and vision was seen as a barrier to transformation:

The mindset of the practice is probably the problem and unfortunately our practice doesn’t really have that mindset about…quality and improvement. (Former PCMH1GPC-L)

Lack of engagement was attributed to “inertia” and resistance to change, and seen as demoralising for those wanting to transform their practice:

It can be quite demoralising, a single voice and everyone else is just not interested in what you want to do (Former PCMH1GPC-L).

Non-engagement with a PCMH vision was also attributed to financial disincentive to change: “If you were a young registrar coming up, why would you do something that’s different?” (Non-PCMH5LC, PP-S). Some non-PCMH participants raised doubt about the value of a PCMH model and expressed concern about government agendas and additional burden for GPs:

The government wants to shift the responsibility to the GPs. It’s cost saving…give it to the GP to do rather than let us just get on with caring for the patient. I don’t see my role as a GP as supervising lots of other allied health professionals (Non-PCMH4HC, PP-S).

### Implementation of Patient Centred Medical Home strategies

This theme gathered participant comments related to implementation of PCMH strategies. These comments highlighted the importance of leadership in driving change and engaging the team; time required for planning and implementing; and PCMH support and training.

Interviewees across all practice types described leadership as critical in driving change; “it’s the principals that drive the change” (Non-PCMH6HC, PP-S). In PCMH transitioning practices, engaged leadership was noted to facilitate team engagement:

Before, you felt it was your responsibility to do it all, but now, you’ve let go a bit more in a team, I think it’s good (PCMH8PP-L).

Where leaders were not engaged, it was difficult to implement PCMH transformation:

…in my position as not as an owner of the practice…as basically a GP that works in the practice…it proved very difficult to actually make changes (Former PCMH1GPC-L).

Many participants commented on the time and effort required for PCMH transformation describing it as a “slower” process than anticipated and requiring perseverance to see outcomes. Several interviewees from PCMH practices described breaking down implementation into manageable components and celebrating small achievements:

…you’re never going to achieve the whole thing in a very short period of time…we need to aim for small achievements and be actually happy about those small achievements (PCMH3PP-L).

Others advised that changes needed to be implemented slowly and commented that to avoid change fatigue they needed “to learn to not try to do too much” (PCMH5PP-S).

Ongoing support and training about PCMH processes, particularly use of data for quality improvement, were valued: “we couldn’t have done it without being educated” (PCMH6PP-L). In-practice support was seen to be “beneficial” particularly if it was “tailored for a specific practice” (Former PCMH1GPC-L). Yet there were also several practice managers and practice nurses who voiced uncertainty about aspects of PCMH, and felt that the training they had received was not relevant to their needs:

How does it work? I’d like a precise guideline of what it’s supposed to be because I still can’t get in my head what exactly is this PCMH (PCMH1PN-S).

Some interviewees from non-PCMH practices who were interested in a PCMH model of care suggested that providing customised support, “a case manager”, “an Internet website or a portal with simple answers” (ICP4PP-L), and guidance from PCMH practices in regards to facilitators and barriers to transformation would be useful:

…getting feedback from other people to see what they found beneficial or what they thought have been drawbacks to the system, so we don’t have the pitfalls (Non-PCMH6HC, PP-S).

Interviewees from all practice types acknowledged the benefits of receiving IT support, and the need for this to be ongoing. However, many participants described challenges with software required for PCMH change and noted the importance of reliable, fully functional IT systems from the beginning of PCMH transition:

We start to launch and see how it’s going and that doesn’t work. You waste lots of time. You create something, you test it and when it is waterproof then you launch it (PCMH1PP-S).

Also highlighted by interviewees from all practice types was the need for staff training in computer literacy, particularly for senior GPs who had a lack of exposure to and experience with IT. Resistance to using computer applications was perceived as a barrier to PCMH transformation with some interviewees noting it “a struggle” to get doctors “more IT savvy”: “The senior doctors are not used to the computer. A lot of them still don’t use it” (Former PCMH2PP-S).

### Structures and processes related to Patient Centred Medical Home transformation

The structures and processes described by interviewees in relation to PCMH transitions included working together as a team; operational changes and new staffing models; staffing and space implications; data driven care utilising information technologies; use of electronic communication with external stakeholders; challenges of the fee-for-service approach, and inadequate funding of new models of care.

Building a multidisciplinary team was considered to be important across all practice types. Interviewees, particularly among PCMH transitioning practices and ICP practices described building teams by bringing in allied healthcare providers and specialists to work in the one location. Non-PCMH practices noted their need for support to help build multidisciplinary teams:

Ideally if you can get like a psychologist or a podiatrist then that would be better…then we can start building that sort of multidisciplinary team around which we can involve our patients (Non-PCMH6HCPP-S).

Practices that intended to transition to a PCMH model suggested the multidisciplinary team “might integrate into the system in a more efficient way” (ICP4PP-L) when it was introduced as part of a PCMH model.

Transitioning practices described a shift to a more nurse-driven model of care, enabling nurses to work at the “top of their licences”:

Doctors will need to have the input obviously, but it will be driven mainly by the nurse (PCMH7PM-L).

The shift to holistic, streamlined, team-based, nurse-driven care in PCMH and ICP practices was described by some as a cultural change from individual (particularly GP) care of patients, to working together as a team, that shares patient care:

What we’re changing is the culture of, “I don’t have to do this alone…I can just rely on my team to do things because it’s our patient” (PCMH6PP-L).

Aligned with this view, some participants noted that when GPs perceived patients to be “theirs”, this limited the role of the practice nurse:

I think the biggest problem that needs to be overcome is the attitude of GPs, that they don’t like other people intruding into their patient care…they don’t trust that their nurse can actually operate within the scope of their licence and do much more. They have this mindset that nurses do dressings and immunisations and that’s it (PCMH3PM-L).

Additional activities incorporated into PCMH staff roles were said to require more time than was available and overburdening staff with new roles posed a risk. Some interviewees recommended there be greater clarity concerning staff roles, particularly in implementing PCMH models of care:

Our front desk staff are already doing maximum with their time. There's no free time for them, so we have to create more time for them to do these extra roles (PCMH5GPC-S).

I don’t actually know what my role will be. What will I be expected to do? Other things that I’m not currently doing? (PCMH1PN-S).

Communication was perceived as essential for effective team-based care with PCMH interviewees highlighting the importance of frequent “huddles” (small group meetings to plan patient care):

…morning huddles, afternoon huddles where we're talking about planning the care with the people here every day, like the nurse and the front desk so that they know who's coming and why they're coming (PCMH5GPC-S).

The term “huddle” was specific to PCMH practices, however, team-based discussions of patient needs and sharing and learning together were also valued among the non-PCMH practices:

Lots and lots of chats going on in corridors and people saying, “what about [name], did you see him the other day”? That sort of informal stuff that keeps the whole connectedness very real…there's no question that there's lots of that stuff happening where at least two people stand together and discuss a patient (non-PCMH9HC, PP-S).

Interviewees from PCMH transitioning practices noted a range of resulting implications for staffing. The need for recruiting committed, like-minded staff with a shared vision was considered crucial, yet there were challenges for practices in maintaining a stable team:

…you need to have all the stars aligned, have people with the right attitudes and the right skillsets coming in to interview and it is really hard. It’s hard to get good applicants and then it’s hard to keep them (PCMH3PM-L).

Interviewees described difficulties in retaining GP contractors and practice nurses, particularly due to the lack of financial incentive to engage in PCMH style practice:

[GP registrars] usually get a better option, better offer. It’s a totally different type, but how on earth can you compete? So it’s very hard to retain (PCMH1PP-S).

Working together as a multidisciplinary team in the one location was reported to require additional space. Many interviewees across all practice types, particularly from solo, small and medium-sized practices described the difficulties with providing the physical space required for PCMH transformation. Some interviewees recommended that space requirements be considered in the initial planning. Others described planning modifications and additions to existing structures. One practice principal was accommodating a larger practice team by purchasing property close to the existing practice:

…we can’t add on, so we cannot give you allied health professional rooms…there’s only two consulting rooms so only two doctors can work at any one time (non-PCMH4HC, PP-S).

We don't have a lot of space to do things, so sometimes that actually limits what we can do in terms of group sessions and things like that (PCMH5GPC-S).

We haven’t got any rooms for any of the allied health personnel so I’m just buying a property next door where there will be larger space…this [PCMH] is what prompted me to do it, because we haven’t got space (ICP5PP-S).

Besides having a lack of physical space, solo and small group practices in particular found it difficult to attract and fund nursing staff and allied health:

It’s not easy to get a nurse for the practice. They used to come and they didn’t have enough work, and they were working part-time, and they wanted a full-time job (Former PCMH2PP-S).

As a solo practice, it’s very difficult for the doctor to employ the practice nurse and bring allied health in the same practice (non-PCMH8LC, PP-S).

PCMH interviewees described the importance of building a well coded electronic medical record to enable review and analysis of patient data. This was stated to be essential for quality improvement. Regular clinical audits enabled PCMH practices to track the health of their patient cohort, see where quality improvement was needed, and plan doctor prompts and patient reminders and recalls:

…improving data quality and then once we've got good data actually analysing it and seeing where we need to improve and then focusing on those areas (PCMH5GPC-S).

Interviewees described using plan-do-study-act (PDSA) improvement cycles addressing key performance indicators (KPIs) to support quality improvement. Some interviewees suggested that data comparisons between practices would help to improve data quality as well as health outcomes:

It’s good to get that feedback about how your data compares to other practices and also how your data compares over time, any improvements (Former PCMH1GPC-L).

Whilst IT was a key enabler for quality improvement, interviewees from all practice types highlighted challenges. The Australian national online health record (MyHealth Record), was described by interviewees as “clunky” to use and time wasting. An electronic care plan shared between the GP and the hospital, was reported to be poorly integrated with GP software, requiring time-consuming manual input of data - “double work for somebody” (PCMH4PM-L). Some practices refused to use it:

one of our major handicaps is our software…the toolbar does not talk to the Linked-EHR and they have to physically put the stuff…lots of GPs in our last meeting stopped using the service because it’s a headache, so I stopped and once I stopped and I don’t use it I forget it (PCMH1PP-S).

These IT challenges impacted on communication with external stakeholders, particularly hospitals:

We still have to call especially…the hospitals. You never get anything from the hospitals…and it’s got to be also in a format where it’s easy to read. You can’t go through a ten-page discharge summary (PCMH8PP-L).

A number of interviewees called for one fully integrated electronic system across primary healthcare systems and hospitals:

We have to just really get a system, one system, or a system that will talk to other systems…but it needs to be real time (PCMH4PP-L).

Others, however, reported that communication had improved with hospital discharge summaries more promptly received:

What is working better is we’re getting lots of referrals from all the hospitals really good now. They come up very promptly, almost immediately. We get them electronically so that actually works really well (PCMH6GPC-L).

The Australian fee-for-service remuneration in general practice was cited as a significant barrier to PCMH transformation, perceived as encouraging throughput rather than quality care. It was described as a poor fit with PCMH models of care, particularly the lack of funding for nursing staff and lack of Medicare funding for non-face-to-face care provision:

In the current fee-for-service model the only way you can generate income for the practice is to see patients and then that really leaves you stuck on the same road (PCMH5GPC-S).

Many interviewees also considered the current HCH trial remuneration to be inadequate and not inclusive of important elements of PCMH, such as costs of non-medical staff and additional registered nurses. The HCH trial was seen by some to be a distraction from implementing a true PCMH model of care:

…if, for example HCH required more time to manage financially and more time to report on it then it actually could be a distraction…it might be damaging to the patient centred medical homes (PCMH5PP-S).

### Outcomes of a Patient Centred Medical Home model

Outcomes attributed to a PCMH model of care included perspectives and experiences from GPs and practice staff of: enhanced patient-centred care and improved patient health; improved provider satisfaction, including through upskilling; and potentially improved health system cost efficiencies, especially through reduced hospitalisations.

Moving towards a PCMH model was described as improving relationships between patients and the practice. Interviewees highlighted PCMH values of patient-centred care where “you put your patient as number one” (PCMH1PP-S). Patients were included as members of the care team and involved in decisions about management of their care. A comprehensive team-based, patient-centred care model, with a focus on preventive care and follow-up was perceived by staff to improve patient/care team relationships. Interviewees also noted anecdotal evidence from patients that this model helped improve patient understanding and satisfaction:

We managed to follow through those patients more effectively than maybe five years before...and the patients are happy about that (PCMH2PP-L).

Interviewees perceived co-location of services as more convenient for patients, enabling them to be seen by multiple care providers at one location and often during the same visit: “the patients find the convenience useful and they certainly like to come to one place” (ICP3PP-S). This reduced waiting times and enabled more efficient care: “A lot more things can be done for the patient a bit more efficiently…if everybody’s on site” (ICP1PP-S).

However, interviewees noted that some patients, particularly the elderly, found the shift to preventive healthcare and team-based care challenging, as it was a perceived threat to their longstanding relationship with their GP. Several interviewees suggested a role for patient education about the PCMH model and services available in primary care:

I don’t think they know of a lot of the avenues open to them…patient education to let them know…just what is available to them and why we see them and do what we do” (PCMH8PN-L).

Working on improving care as part of a team improved job satisfaction for interviewees from PCMH and ICP practices. There were reports of staff “very keen to be upskilled” (PCMH5GPC) and examples of multiskilling and career progression:

It's improving their job satisfaction and involvement in the whole team because they actually become part of the team (PCMH5GPC-S).

We’ve got our senior receptionist going through the medical assistant course…learning about cholesterol targets and blood pressure targets and how often all these different tests should be done (PCMH3PM-L).

Practice nurses were enabled to use their skills to best advantage, for example through following up patient care plans:

Most of the care will be done by the nurse…so we promote them and say “practice nurses this is what you do and you are a carer and you are a clinician and you help us” (PCMH2PP-L).

The PCMH model was perceived by most interviewees as likely to save health systems costs and improve outcomes by enhancing efficiency, and reducing hospital admissions:

My absolute conviction is that we already save so much money because we just don’t have patients go to hospital (PCMH4PP-L).

The holistic care provided in the PCMH model was also perceived by interviewees as resulting in positive patient outcomes. A shift to preventive care with effective follow-up was noted and computer software aided PCMH practices to be proactive in providing healthcare:

…we take great pride in making sure a patient’s immediate problem is dealt with, their preventative health is dealt with, seeing what happened in the last consultation is dealt with and also formalising that in a kind of reminder list (PCMH8PP-L).

Proposed patient registration was perceived to provide a means of reducing duplication of medical testing:

We have so much doubling…ultimately the health dollar will be much less because the patient will be restricted to three or four doctors (Non-PCMH1HC, PP-S).

## Discussion

Our findings provide insights into experiences and perceptions of health professionals about PCMH transition across a range of general practices in western Sydney, Australia. Factors facilitating PCMH transformation were having a shared vision, engaged leadership and clear communication within the practice team. The majority of participants across all practice types perceived the PCMH model as likely to facilitate improved patient outcomes by increasing efficiency, reducing duplication of care, avoiding hospital admission, and thereby reducing health systems costs. Job satisfaction was enhanced in transitioning practices through team work and opportunities to upskill. Challenges included recruitment and retention of staff, particularly those with shared values and vision. Many practices highlighted a lack of physical space to implement PCMH strategies and in particular, interviewees from solo and small group practices described a lack of capacity to employ practice nurses and allied health. Many interviewees also commented on the challenges of implementing PCMH approaches within the current Australian funding model.

Transition processes described by PCMH interviewees aligned with the 10 building blocks of high-performing primary care [8], particularly the foundational elements of engaged leadership, team-based care, and data-driven improvement. Consistent with international literature, our findings indicated that engaged leadership was imperative in driving PCMH transitions [6, 7, 42, 43], and that team-based care was key to the model, requiring protected time for meetings to plan patient care [6, 42, 44]. Conversely, lack of leadership and an absence of staff engagement, particularly among former PCMH interviewees, were cited as barriers to transition, as reported in other studies [7, 45].

A clinical audit tool enabled data driven care, highlighting where improvement was needed, however, IT challenges were cited by the majority of interviewees as a major barrier to quality improvement, in common with other studies [7, 42, 45]. Interviewees noted that IT systems required time and effort to establish and data entry was often duplicative.

The PCMH model was perceived by interviewees from PCMH transitioning practices as addressing the quadruple aim [11] - improving the health of the population, patient and healthcare provider experience, and reducing costs. The model was reported to have the potential to improve population health through use of data to track health outcomes. Interviewees described patient satisfaction with a focus on preventive care, where patients were included in making decisions about their health, and were able to access a range of health services in the one location in a single visit, thereby improving accessibility and reducing patient costs. Interviewees in PCMH practices enjoyed working as part of a team and valued opportunities to learn new skills and try out new roles. Consistent with other research, these factors were stated to improve work satisfaction, and create a sense of achievement through professional and personal growth [46, 47]. The model was also suggested as to be likely to reduce health systems costs, through reduced duplication of tests and hospital admissions.

The current Australian fee-for-service funding of general practice was seen by interviewees across all practice types as a disincentive to PCMH implementation and a poor fit with PCMH and HCH models of care. The lack of funding for PCMH strategies such as non-face-to-face consultations and team-based care was of concern, particularly for PCMH transitioning practices, as was the perceived inadequate funding for chronic disease patients under the HCH model. These findings highlight the need for funding reform, including payment for non-face-to-face care, care coordination, and population-based care as advocated widely [10, 20, 48, 49].

Our interviewees highlighted the valuable support provided by Western Sydney PHN, including PCMH education and practice visits, assistance with quality improvement activities and IT training. The key role of the western Sydney PHN in PCMH transformation exemplifies the value of a regional organisation funded to support local innovation in healthcare [12, 48, 50, 51].

### Strengths and limitations

Whilst the real world context and the variety of participating practices were strengths of our research, we explored practice transitions which remain a work in progress. Our findings therefore may not present a complete picture of the entire transition experience. Our research focussed on one region in Australia, however our findings on experiences of the PCMH change process can inform both Australian and international primary health clinicians and researchers concerning facilitators and barriers to PCMH implementation. Our research was limited to GP and practice staff perspectives on PCMH transition. Patient perspectives were not included in the current research, however investigation of the views of these key stakeholders would provide a valuable contribution to understanding the challenges and impact of practice transformation. Future research on PCMH transformations should also include trials comparing health outcomes and costs related to these transitions as well as health systems cost savings related to this new model of care.

## Conclusion

Patient Centred Medical Home models of primary care have the potential to improve quality, cost-effectiveness and equity of health outcomes in Australia as in other countries. Based on our findings we recommend strategies for practices transitioning to these new approaches to primary care. These include strong leadership from principal GPs to promote PCMH values and vision, and engagement of the whole practice team in implementing patient-centred, data-informed, team-based care with a registered patient cohort. Patient Centred Medical Home transition requires development of new structures, processes and a different culture, as well as training and tailored support for all staff, and adequate infrastructure, especially information technology. To support PCMH transitions in the Australian context there is a need for funding for non-face-to-face consults and for enhanced nursing and allied health roles. Crucially, health system policy changes, including primary healthcare funding that remunerates quality practice rather than patient throughput would facilitate practice transition to PCMH models of care with their likely benefits.

## Supplementary information

**Additional file 1.** Semi-structured interview guides.

**Additional file 2.** PCMH thematic analysis.

**Additional file 3.** COREQ Checklist.

## Data Availability

Raw files are not available due to privacy reasons. Findings are reported in the paper in the form of quotations. The full thematic table of participant quotations and the interview guides are included as supplementary files.

## Abbreviations

EHR: Electronic Health Record
GP: General Practitioner
HC: High Capacity
HCH: Health Care Homes
ICP: Integrated Care Program
IT: Information Technology
KPI: Key Performance Indicator
LC: Low Capacity
PCMH: Patient Centred Medical Home
PDSA: Plan, Do, Study, Act
PHN: Primary Health Network
RACGP: Royal Australian College of General Practitioners
SEIFA: Socio-Economic Indexes For Areas
WSICP: Western Sydney Integrated Care Program
