# Supplementary material for: Patient Centred Medical Home (PCMH) transitions in western Sydney, Australia: a qualitative study
Source: BMC Health Serv Res. 2020 Apr 6;20:285. doi: 10.1186/s12913-020-05123-7 (PMC7137239; doi:10.1186/s12913-020-05123-7)
Supplement: Supplementary file 1 — Additional file 1. Semi-structured interview guides. [file 12913_2020_5123_MOESM1_ESM.pdf]

## Additional file 1: Semi-structured interview guides

**PCMH practices**

1. In what ways has your practice changed since starting the PCMH project? How would you describe (tell me about) your PCMH transformation progress?

2. In what ways is the progress your practice made what you had anticipated?

Thinking about elements or practices that the practice has put into place:

   a. What helped make this happen?

      i. *Probes*: leadership, cultural changes, operational changes, resources provided by WW, learning orientation/QI processes, structural characteristics of practice, conditions in external environment

   b. What problems had to be overcome?

      i. Probes: leadership, change management and change fatigue, information systems, operational barriers like scheduling, competing priorities, lack of time, patient resistance, payment systems, structural characteristics of practice, conditions in external environment

3. In what ways is the progress different from what you had anticipated?

   a. Why do you think this happened?

4. How have the structural, technological or other changes associated with becoming a PCMH assisted or interfered with the provision of effective, patient-centered clinical care?

5. At this point, what is your vision for your practice as a PCMH? (Goals, important next steps)

   a. Do you think your practice will be able to achieve these goals and make these steps? Why or why not? What would help you achieve these goals?

6. [*If practice has applied to be a HCH*] What difference do you think the Health Care Homes demonstration will make for your PCMH journey?

7. What are 1-2 things you know now that you wish you had known when you started? (*Or phrase as advice to colleague considering PCMH, or advice for someone designing a PCMH initiative*)

**Former PCMH practices**

1. Can you tell me about your past work on PCMH? What sort of practice transformation did you work on while involved in the PCMH learning collaborative?

2. What are some successes you had with implementing a PCMH model? Which elements or practices were you able to put into place?

    a. What helped make those happen?

        i. *Probes*: leadership, cultural changes, operational changes, resources provided by WW, learning orientation/QI processes, structural characteristics of practice, conditions in external environment

3. Are those elements still in place? Why or why not?

4. What aspects of the PMCH model were more difficult to start or establish?

    a. What problems had to be overcome?

        i. Probes: leadership, change management and change fatigue, information systems, operational barriers like scheduling, competing priorities, lack of time, patient resistance, payment systems, structural characteristics of practice, conditions in external environment

5. What are 1-2 things you know now that you wish you had known when you started? (*Or phrase as advice to colleague considering PCMH, or advice for someone designing a PCMH initiative*)

6. [*If hasn't already been answered*] What are the reasons for your practice leaving the PCMH group? For stopping active work toward a PCMH-type model?

7. [*If relevant / not already answered*] At this point, does your practice have any interest in formally pursuing a model home model? Why or why not?

    a. Is there any sort of assistance or resources that would help if you did?

**Non-PCMH practices (ICP and others)**

1. The idea of the patient-centered medical home is getting a lot of press these days. What is your understanding of what a PCMH does, or looks like?

2. From your perspective, what would be the benefits of a PMCH model for you, for your practice, and for your patients?

3. What would be the costs or drawbacks?

*For interviewees who have some knowledge of PCMH concepts …*

4. Does your practice have any interest in pursuing a model home model? Why or why not?

   i. Probes: compatibility with their practice culture or structural characteristics?

5. Do you think your practice would be able to become a patient-centered medical home?

   a. What might be the obstacles?

   b. What would help?

*For interviewees who say they have very little knowledge of PCMH …*

6. Tell me about what it's like, from your perspective, to work in this practice.

7. What are some good things about how this practice functions?

8. What are some things that could be changed or improved?

*For both …*

9. How does change happen in this practice?

   a. Please give me an example of how something in the practice has changed in the last 6 months.

   b. Who are the people in the practice who can really make things happen on changes that people want to see?

**Sources**

Alidina at al. (2014). Practice Environments and Job Satisfaction in Patient-Centered Medical Homes. *Ann Fam Med* 12(4): 331-337.

Goldman at al. (2015). Recommendations for a Mixed Methods Approach to Evaluating the Patient-Centered Medical Home. *Ann Fam Med* 13(2): 168–175.

Helfrich et al. (2016). The facilitators and barriers associated with implementation of a patient-centered medical home in VHA. *Implementation Science* 11:24.

Janamian et al. (2014). A systematic review of the challenges to implementation of the patient-centred medical home: lessons for Australia. *MJA* 201(3): S69-73.
