# Supplementary material for: Patient Centred Medical Home (PCMH) transitions in western Sydney, Australia: a qualitative study
Source: BMC Health Serv Res. 2020 Apr 6;20:285. doi: 10.1186/s12913-020-05123-7 (PMC7137239; doi:10.1186/s12913-020-05123-7)
Supplement: Supplementary file 2 — Additional file 2. PCMH thematic analysis. [file 12913_2020_5123_MOESM2_ESM.docx]

# Additional file 2: PCMH thematic analysis

The following analysis of the interview data is structured within four main categories of: Vision; Implementation; Structures and Processes; and Outcomes. The key themes corresponding to these categories are provided in the table below. In the analysis, each of these key themes is further elaborated by subthemes which are then described and illustrated by selected quotes.

| **Vision** | **Implementation** | **Structures and Processes** | **Outcomes** |
| --- | --- | --- | --- |
| Alignment of vision | Leadership | Working together as a team | Patients |
| Engagement in realising the vision | Management | Staffing implications | Practices |
|  | Support and training | Data driven care | Health systems and costs |
|  |  | Information technologies |  |
|  |  | Communication with external stakeholders |  |
|  |  | Fee for service |  |
|  |  | Uncertainty regarding PCMH and HCH models |  |

All participants’ voices have been presented in the analysis. Colour coding is provided to identify the type of practice and role of participant within the group. The following key provides relevant colour codes.

**Participant Key:**

PCMH

Former PCMH

ICP

Non-PCMH - higher capacity

Non-PCMH - lower capacity

| PCMH Vision | |
| --- | --- |
| Alignment of Vision | |
| - *Alignment of values prior to PCMH concept*      - *Activities are aligned to values* - *Alignment of values helps enable transition to PCMH model of care* - *PCMH vision informed by quality improvement* - *Commitment to continuing with PCMH model* - *Lack of staff alignment to PCMH values and vision a barrier* - *Corporate lack of commitment to the vision* - *Doubt about value of PCMH model among Non-PCMH practices* - *Concerns about government agenda* | *their philosophy lines up very nicely, it’s very congruent with the patient-centred medical home. People were already on the page, they were already aligned with those sorts of goals. PCMH 4, Practice Manager  *even before this concept was there we always felt that this practice has to be a medical practice not a medical centre and we always wanted to know our population and we were not happy with a more highly transient population, so we wanted our patients to remain with us. PCMH 2, Practice Principal B  *we were already doing a lot of the things and trying very hard to achieve some of the changes that are already part of the building blocks in terms of data management, engagement with patients, registration, having people choose their GP, trying for continuity. PCMH 4 Practice Principal  *****we came together with exactly the same philosophy and it’s that like-mindedness that in the end has allowed us to begin the process in this practice PCMH 4, Practice Principal  *Patient centred team-based care that is data driven and continuous quality improvement, continuous. We will not stop changing to improve. That’s the vision PCMH 6, Practice Principal  *We three directors had a vision. I mean, we wanted it to happen anyway so whether this is going to rollout or not, we are going to do that anyway PCMH 2, Practice Principal A  *the mindset of the practice is probably the problem and unfortunately our practice doesn’t really have that mindset about, as much as you’d like to be into the quality and improvement Former PCMH 1, GP Contractor  *there’s no-one in [corporate] who has I think the necessary degree of interest in the PCMH concept to champion it…the focus is on business process rather than patient outcome PCMH 3, Practice Manager  *I know that [name of GP] wasn’t really happy with the patient centred home medical, so I don’t know whether he’ll stay or not. [Name of another GP] really wasn’t too crash hot with it either PCMH 1, Practice Manager  *the patient centred home is really I think a surrogate just like it was when Medicare was introduced…your ‘home’ which is a very nice little euphemism. Non-PCMH 5, Practice Principal  *I see it as sort of dumbing down how people will ultimately be looked after, if it’s not handled properly. I am yet to be persuaded that it will have a benefit. Non-PCMH 5, Practice Principal  *it sounds to be quite controlling and people don’t like that in Australia. Non-PCMH 4, Practice Principal  *The government wants to shift the responsibility to the GPs. It’s cost saving…I don’t see my role as a GP is supervising lots of other allied health professionals. Non-PCMH 4, Practice Principal |
| Engagement in realising the Vision | |
| **Motivation**   - *Engaging staff in understanding reasons for transitioning to a PCMH model* - *Engaging staff in a common purpose* - *Involving staff in decision-making*      - *Engaging with staff builds trust* - *Engagement encourages staff to take on more roles* - *Lack of financial reward a disincentive to engage with PCMH vision* - *Inertia and resistance to change is a barrier to engaging with PCMH vision* - *Staff disengagement with PCMH vision is demoralising* - *Staff disengagement with PCMH makes transition difficult* | *encouraging everyone to understand why we're doing this makes a big difference. PCMH 5, Dietician  *****So having the team engaged and making them understand why - not just what we're doing but why we're doing it and getting the buy-in is really important to making sure that they feel that their role is important, it's not just pointless or mindless, it's got benefits for themselves, for the patients. PCMH 5, GP Contractor  *if we can explain why we’re doing this and embed that into the belief system of the staff then they will follow. If they can’t see the why, no. PCMH 6, Practice Principal  *for the team to work together the team has to have a common purpose, so we're all working to the same purpose PCMH 5, Practice Principal  *****The key thing is to actually have your team and colleagues be engaged from the onset. PCMH 3, Practice Principal  *it puts a lot of pressure on them [staff] and so they have to understand the why and why we’ve asked them to do what they’re doing, be involved in the decision-making process and have a basically a similar direction. PCMH 6, Practice Principal  *the key element is the trust between team members which allows us to share the care much, much more effectively and ensuring that there was enough respect for everyone to be able to share their views PCMH 5, Practice Principal  *Morning and lunch huddles, which also I think help the team at two levels - one of them is organisation but the other one is, again, building that trust and that familiarity with modus operandi. PCMH 5, Practice Principal  *good staff engagement, that is really helping, and that has become evident through the different members of the team taking on more roles. PCMH 5, GP Contractor  *The other GPs were very, I guess, demotivated by the Medicare freeze and they’re thinking, “well you know, I’m not getting paid for doing this, why should I?” PCMH 3, Practice Manager  *another registrar was here for six months. At the end of the day if you are a young doctor coming out of uni with a big HECS debt why would you go into this? PCMH 1, Practice Principal  *If you were a young registrar coming up, why would you do something that’s different? It might ultimately be useful to people, but why would you do it if you can generate a reasonable income seeing patients at ten minute  intervals? Non-PCMH 5, Practice Principal  *When I wanted to try and expand and get somebody in, I had to deal with the manager…he was trying to squeeze more money out. Non-PCMH 5, Practice Principal  *based on what I’ve found from the owner of the practice the actual financial side of things would be the primary driver of it. It would have to be a good financial case for him to actually be willing to make changes. Former PCMH 1, GP Contractor  *****I’m happy the way things are. As I’ve told you I’ve been practising nearly 40 years now. Former PCMH 2, Practice Principal  *****there’s always been that resistance to change or doing anything about it…the problem is a lot of people they initially like the idea but then actually well when it comes the time to do something, then people go missing and don’t really want to. Former PCMH 1, GP Contractor  *I’ve pushed and pushed and pushed, but it was really not easy, because the other GPs weren’t interested. The rest of the practice are very happy to be left alone and that’s sad but true because there’s a lot of inertia…I did not envisage all of the resistance that I encountered. PCMH 3, Practice Manager  *I think physicians are very difficult. They will be resistant to change. I'm not talking about this [PCMH] as a small change, it's a huge change. ICP 4, Practice Principal  *****when you’re actually trying to do things and you meet a lot of resistance…it can be quite demoralising, a single voice and everyone else is just not interested in what you want to do Former PCMH 1, GP Contractor  *So if there are people in the medical centre who are against the idea, who’s not coming along with the ride it just makes the process much harder so that would definitely be the most critical part. PCMH 3, Practice Principal |
| Implementation of PCMH strategies | |
| Leadership | |
| **Driving change**   - *Leading with authority to drive and implement change* - *Leading and driving change from the top* - *Motivation to change is patient well-being* - *Involving the team* - *Strengthening team engagement through leadership* - *Making changes without leadership is challenging* - *Leadership training is required* | *obviously in a practice to actually implement change you need to have leadership Former PCMH 1, GP Contractor  *it’s the principals that drive the change, if we’re passionate about a particular topic, well we’ll go out and do things. Non-PCMH 6, Practice Principal  *I guess [name of Practice Principal] has been the driver behind this a lot. Having somebody who actually can lead and drive the change makes a big difference, but if there is no one driving it or not passionate enough to make the change then it's probably going to be difficult PCMH 5, Dietician  *People generally don’t like change and it is just that, it has to be driven from the top. PCMH 7, Practice Manager  *I'm accused of being a bit autocratic. I think I need to set an example… Non-PCMH 10, Practice Principal  *I can just rely on my team to do things because it’s our patient. We are going to look after the patient, not I am going to look after the patient. PCMH 6, Practice Principal  * Before, you felt it was your responsibility to do it all, but now, you’ve let go a bit more in a team, I think it’s good. PCMH 8, Practice Principal  *****there needs to be that engaged leadership. If you're floundering around and you don't know where you're going there's no point trying to make people follow you. You've got to be pretty committed to the medical home model and engage your team and give them reasons for why they want to be part of it. PCMH 5, GP Contractor  *in my position as not as an owner of the practice…as basically a GP that works in the practice and not having the sort of actual leadership, actively engaged in the actual progress, it proved very difficult to actually make changes Former PCMH 1, GP Contractor  *before you start the process you really need to do a little bit of psychology, a little bit of research into your leadership style and the behavioural styles of the people that you’re going to be working with. PCMH 4, Practice Manager  *****they haven’t had any leadership training or any experience in that area…there’s no focus and there’s no learning about leadership, so it’s a huge paradigm change for a lot of GPs. PCMH 3, Practice Manager  *They need to be able to encourage and negotiate and do conflict resolution and there are just so many skills that a lot of GPs simply don’t have. PCMH 3, Practice Manager |
| **Engaging Team**   - *Importance of respect in staff engagement* - *Enable each member of the team to contribute* - *Including and involving all staff in team-based care* - *Engage team in seeking innovative solutions and approaches* - *Team building crucial for implementing PCMH changes* | *You have to engage the entire team and if you’ve got people who are very direct and brusque in their communication style, they’re going to get nowhere PCMH 3, Practice Manager  *ensuring that there was enough respect for everyone to be able to share their views and everyone felt comfortable to contribute to the care of a patient, such that actually each team member was able to step up and say, yes, I'll take that, or that one I'll do, or I'll call this person. PCMH 5, Practice Principal  *you need everybody on every level to know what it’s about and be engaged in it and actively engaged. So that involves from the front desk, from the second they walk in really because, if the front desk staff don’t know what being a patient-centred medical centre is, then they will have no idea why the people are coming in or how to recruit people to the systems. PCMH 7, GP contractor  *the first change is culture and a shift in mentality of the staff from “my patient” to “our patient”. Now, we work as a great team PCMH 6, Practice Principal  *So involving other members of the multidisciplinary team - that would be the dietician, exercise physiologists, everybody along those lines - really getting them more involved in the patient care. So we've been able to improve our team-based approach PCMH 5, GP Contractor  *how are we going to address it as a group? And then it might be that's where other solutions like group education sessions or small group education sessions, shared medical appointments come into it, joint consultations for very intensive care for some patients. PCMH 5, Practice Principal  *if you’re not building a team that can help you with these sort of changes, it’s not going to go very far unfortunately. Former PCMH 1, GP Contractor |
| Management | |
| **Planning and Implementing**   - *Planning and implementation is a slow process* - *Time taken in using a team based approach* - *Time and effort needed to enrol patients* - *Time taken for change to be adopted* - *It takes time to see any benefits* - *Not as straight forward as anticipated* - *Implementing by breaking down into workable components* - *Small achievements building blocks for future success* - *Learning as you go* - *Need for perseverance* - *Respecting the process by not doing too much at a time* - *Implementing too much at once is confusing and can slow down the process* - *Concentric circle model for change process* - *Keeping the vision at the centre* - *Difficult to implement changes without a team* - *Need for keeping a big picture approach* - *Implementation process different for each practice* - *Risk of loss of interest for non-PCMH practices if change takes too long* | *that took us a while to actually have the thought about what a medical home could look like. PCMH 5, Practice Principal  *I think the stuff is consuming a lot of time. PCMH 1, Practice Principal  *There is a lot of changes. I think we’re trying to get them to happen but it’s been slow. PCMH 6, GP Contractor *it’s just so slow and that’s partly because of the amount of work it takes to have those team meetings and you can devote a whole hour to one complex patient…every patient that was enrolled, required a lengthy meeting to look at goals and parameters and targets PCMH 4, Practice Manager  *I thought once I identified patients you add them to the reminder system…and it should run all right but that actually takes up a lot of time and effort from not just myself but also from the team. PCMH 3, Practice Principal  *just the amount of planning and execution, I think that’s more than what I anticipated. PCMH 3, Practice Principal  *it’s just taking a lot longer, the transition, than I suppose a lot of us thought it would PCMH 6, GP Contractor  *I didn’t realise it takes so long. We’ve been trying to do this for two years. It takes a hell of a lot of time to change things. PCMH 6, Practice Principal  *I think the progress is even slower than I had anticipated. I had anticipated it would be slow, but it’s just so slow PCMH 4, Practice Manager  *when the change comes you don’t immediately realise that this is a benefit. It takes time. PCMH 2, Practice Principal A  *I think it's probably not as quick or as straight forward as you would anticipate PCMH 5, GP Contractor  *carefully looking at the building blocks and breaking them down into a plan and then going back and reassessing everything after you've done it to see what's actually helping and what's not necessary, what are you doing that's working and what's not. PCMH 5, GP Contractor  *we can trial and error, we can see how that works and how to fine tune the system but then we can keep rolling from there. PCMH 3, Practice Principal  *the only thing is take it one step at a time because you’re never going to achieve the whole thing in a very short period of time. So we need to aim for small achievements and be actually happy about those small achievements and as we keep building on that it will gradually get bigger and bigger. PCMH 3, Practice Principal  *It may not happen the first time, but maybe the second time or third time. So, it is a slow change process, but you just have to keep going on. PCMH 7, Practice Manager  *we actually had to learn to not try to do too much. We had to actually learn to respect it…we can't do too many changes. It has to be focused. PCMH 5, Practice Principal  *It's trying to work out how much you can reasonably do at one time because if you try to do too much everyone just gets confused. PCMH 5, Practice Principal  *Make sure everyone understands the why before you start because we went six months ahead, way ahead of the rest of the staff and we forgot to look behind to see who is coming along. PCMH 6, Practice Principal  *There was probably perception at the beginning that it was a step-wise approach and it was a very iterative approach, whereas it's actually not. We feel that it's very much more and the term which I like to use is the concentric circles approach. So you have at the very, very centre your vision, but around that you have a number of things which flow from that vision. PCMH 5, Practice Principal  *You need to keep a big picture view on it from the Practice Principal or from the senior management side. This is the vision that we see…sometimes people get so task oriented that they just fail to see the bigger picture. PCMH 7, Practice Manager  *You’ve got to partner with someone because you can’t do it on your own…when I’m feeling demoralised and down and exhausted [name of Practice Principal] is rearing to go and vice versa, we encourage that, I don’t know how anyone could do it on their own. PCMH 4 Practice Principal  *You need to have a good team with you because it's hard to do it on your own PCMH 5, Practice Principal  *You need to work with them because you can’t do it alone. PCMH 3, Practice Manager  *I would say that there is no definite right or wrong way. I think there is a general broad direction that you go in but the specifics are probably going to be different for each practice. PCMH 5, Practice Principal  *I think it's taking too long. We are convinced, it has been explained, everybody knows there is need, but it is not happening…it has to be done efficiently and fast…It has to happen quickly because if it takes too long people will lose interest. ICP 4, Practice Principal |
| Support and Training | |
| **PCMH education and workshops**   - *PCMH education is essential* - *Opportunity to trial PCMH components useful* - *Meso-organisation leadership enables a big picture approach* - *Help needed for training to be implemented into practices* - *Help needed for training to be tailored to individual practices* - *Need for practice managers and nurses to have a clear understanding how PCMH model operates* - *Need for PCMH training to be relevant to staff needs* - *Need for the provision of PCMH information, training and support for Non-PCMH practices* | *we couldn’t have done it without being educated – all the education we have done on this. All the group work we done on this. All the meetings that we have had. All the leadership skills that we’ve learnt. All the data skills that we’ve learnt and the tools that we’ve been given by WentWest to do all of that. There’s no way on earth that we could have done it independently, just no way. PCMH 6, Practice Principal  *WentWest support definitely makes a big difference because the big picture of PCMH practice, there are lots of components and each component it takes a bit of inertia for it to get going and a bit of trial and error, so the program that Went West does to get us to trial the components actually do help a lot PCMH 3, Practice Principal  *It helps us when we have an organisation like WentWest supporting us because they can take that big picture approach, where they can sort of give us information, hold sessions. PCMH 7, Practice Manager  *there was a lot of good information that was available that we heard about but the problem was always taking the information and then actually implementing it in our practice and all. Former PCMH 1, GP Contractor  *in workshops you get overall ideas and the thoughts about how the sort of changes can be made, but often it’s on the more individual basis is the best way to have things that are tailored for a specific practice Former PCMH 1, GP Contractor  *I don’t even know what are these changes that we’re supposed to be implementing? How does it work? I’d like a precise guideline of what it’s supposed to be because I still can’t get in my head what exactly is this PCMH? How is it supposed to run? Show me a video of a practice that has it in place working, how they recruited the patients, from the very – step-by-step. PCMH 1, Practice Nurse  *Honestly, they were boring. They were all day and time consuming [PCMH workshops]…I don’t understand why we’re sitting here colouring in something or drawing something. It’s not relevant. Half the time we don’t understand what they’re talking about PCMH 1, Practice Nurse  *I don’t have any idea about what it [PCMH] is exactly. [Name] who’s the supporting worker for the GPs from WentWest, he came and discussed about that but he hasn’t given me much information on that…so I’m really keen and interested but I want to know more about it - How it is going to help the patients and how it’s going to include our delivery of the care as well. Whatever we’re doing is it going to improve their care more or it’s going to give problems more? ICP 2, Practice Principal |
| **Support from PHN**   - *Individualised support from PHN valued* - *Visits from PHN useful for tailoring support* - *Ongoing PHN support critical to change process* - *Need for practical advice on PCHM operations* - *Need for feedback to guide new transitioning practices* - *Need for easy access to support e.g. through web based portal* | *WentWest actually played an important part because finally we have one person to talk to when we have some issues. Non-PCMH 1, Practice Principal  *I have had [name] come from WentWest, actually she did speak about the [PCMH] model, what it’s going to be. Non-PCMH 8, Practice Principal  *WentWest of course is hovering all the time, offering stuff for us, and we call on [name] or whoever it is who's currently our local person. Non-PCMH 9, Practice Principal  *We need her [WentWest staff], yeah. We constantly…we’re always ringing her up – very good resource, like, absolutely fantastic…she’s become a member of the family here PCMH 1, Practice Nurse  *it’s more that on the ground support, in terms of practice support in person that often is beneficial because you want to be doing things…that are tailored for a specific practice Former PCMH 1, GP Contractor  *She was here from about 9 o’clock until about 2 o’clock helping our practice nurse do some of the Linked-EHR care plans. That was fabulous and you know people really appreciate that help because otherwise they feel like they’ve just had a lot of work thrown at them and they don’t see the point to it. PCMH 4, Practice Manager  *she [WentWest worker] always has an agenda when she comes here on a Tuesday, we’re going to do this today we’re going to look at this data. PCMH 1, Practice Manager  *WentWest’s involvement is very, very important because if that stops then it won’t happen. Without their involvement, support, guidance I don’t think we would be able to achieve 10% on our own of what we have done. And they work very well, like in the sense that they also acknowledge when practices are going through a busy time but then they also keep coming and – which I think is really great, they keep coming back. They don’t give up. No. PCMH 7, Practice Manager  *maybe just some practical advice on how to sort out the pods and look at caring for patients that way…I think it would be very helpful for the doctors to actually see practically how things work if you’re trying to push a patient centred medical home. PCMH 6, GP Contractor  *getting feedback from other people to see what they found beneficial or what they thought have been drawbacks to the system, so we don’t have the pitfalls…a brainstorming type thing from some more experienced practices might be beneficial to us. Non-PCMH 6, Practice Principal  *Our feedback has to be appreciated and it has to be incorporated into the decision-making process ICP 4, Practice Principal  *we must have one case manager or something which we could talk, or an Internet website, or a portal with simple answers to get the things done, to tell us how to move forward. When we are linked to that portal, it will take us to that highway to reach the end. ICP 4, Practice Principal |
| **IT training and support**   - *Functional systems required from the start* - *IT support and training provided* - *Low level of GP computer literacy* - *Staff not confident in IT and need more support* - *Resisting IT is a barrier to implementation of PCMH* - *Training on how to access Linked-EHR care plans* | *****that’s the problem with the software, they don’t bring it out when it’s 100%, they bring it out and fix the bugs as it goes along. PCMH 1, Practice Manager  *over the years we start to launch and see how it’s going and that doesn’t work. You waste lots of time. You create something, you test it and when it is waterproof then you launch it. PCMH 1, Practice Principal  *it [Top Bar] started late 2015, so those first few months I had someone coming out like every week to try and fix every single problem because there were just so many PCMH 8, Practice nurse  *making sure that the IT system doesn’t break down all the time, that would really help. PCMH 2, Practice Principal A  *WentWest I mean, they’re supporting us a lot…they come and do any I.T. issues and they work with our I.T. person. PCMH 2, Practice Principal A  *the WentWest practice support officers, they helped me, so I learnt basically the Medical Director program. ICP 1, Practice Principal  *we have our IT guys and then WentWest. We’ve got some fantastic people from WentWest coming in and helping us. PCMH 2, Practice Principal B  *[WentWest staff] has come around and tried to tell the doctors, but it just goes right over their heads and if it’s not working they always get frustrated and won’t use it. PCMH 1, Practice Nurse  *If you’re not good at a computer, you can’t do this. PCMH 8, Practice Principal  *The IT is a big issue because we don’t know much these days…when we expand there will be a lot of things and we are not computer literate. ICP 5, Practice Principal  *the senior doctors are not used to the computer. A lot of them still don’t use it. Former PCMH 2, Practice Principal  *getting the doctors on board to get them more IT savvy so it’s been a bit of a struggle getting them to change the way they are doing things…there’s a lot of clinician inertia with that. PCMH 2, Practice Principal B  *we had a senior colleague here who wasn’t into IT. He did not want to have anything to do on a computer. He said, “I’ve worked there for 40 years, all on paper”. PCMH 1, Practice Principal  *more encouragement to the doctors, some of them are not so confident to do that [IT tasks]. So a lot of them have gone through medical school without the use of computers…getting them to rely a bit more on electronic health and showing them that’s the way to go. PCMH 2, Practice Principal A  *the doctors don’t even know where to go and find the care plans from the Linked-EHR, where to go, what’s in there, to review them, to update them, I mean, they don’t know how to update their normal care plans. PCMH 1, Practice Nurse |
| Structures and Processes related to PCMH transformation | |
| Working together as a team | |
| **Operational changes**   - *Changing the culture to one of working together as a team* - *Building a multi-disciplinary team* - *Non-PCMH practices note need for more allied health and specialist health professionals in team* - *Staff taking on new roles* - *Planning care across the team e.g. through “huddles”* - *Using GP-patient relationship to introduce new team members* - *Streamlined, appropriate care enabled through team work* - *Team members are valued* - *Practice nurses taking on more responsibility* - *Ownership of patient a challenge for some GPs* - *Need for clarity of staff role and purpose* | *what we’re changing is the culture of, “I don’t have to do this alone” and this particularly stands for the doctors, because we as doctors are trained to be a lone ranger…”I’m responsible for everything and therefore I need to do everything myself”. That’s the biggest change…I can just rely on my team to do things because it’s our patient. “We are going to look after the patient, not I am going to look after the patient”. PCMH 6, Practice Principal  *I prefer to use the term "we,” because I see it as a team basically in a medical centre. ICP 4, Practice Principal  *The term team does tend to help a bit in trying to capture the word…apart from having a focus on patient outcomes, there's also that working together as a group part of it that it's not each person here working in isolation, it's a lot of cross-fertilisation that goes on. Non-PCMH 9, Practice Principal  *from what I understand, it’s like a team work, you try and involve everyone. You have a practice nurse, you have some allied health people here and we try and care for the patients as a whole team rather than being one GP caring for them ICP 5, Practice Principal  *Under one roof we have a multi-disciplinary team…so the patient, the centre, then you’ve got the doctor, the nurse and all the allied health. PCMH 2, Practice Principal B  *we have a good team including the doctors, the GPs and we have specialists, both paediatric and adult specialists. We have allied health - we have physiotherapist, dietician, podiatrist, psychologist, speech pathology and OT as well. So not many practices have speech and OT. We have quite a few paediatricians as well with us. ICP 2, Practice Principal  *We’re slowly increasing our allied health numbers. We have a dietitian once a fortnight, we’re just getting a diabetic educator once a fortnight and ideally if you can get like a psychologist or a podiatrist then that would be better…then we can start building that sort of multidisciplinary team around which we can involve our patients Non-PCMH 6, Practice Principal  *If I were able to wave my magic wand, I would have more allied health people working with me, particularly practice nurses. Non-PCMH 5, Practice Principal  *We are trying to see if we can have specialists working here. We’ve got already three on board… hopefully we can grow to like endocrinologist in the future because this demographics needs one definitely, allied health professionals. Non-PCMH 3, Practice Manager  *two of our front desk are taking on the data managing side of things. PCMH 5, GP Contractor  *morning huddles, afternoon huddles where we're talking about planning the care with the people here every day, like the nurse and the front desk PCMH 5, GP Contractor  *our model here would be that the reception staff or office staff and the doctors look after the patient as a team. Non-PCMH 9, Practice Principal  *Say, “my name is X, I’m Doctor [name’s] nurse. He has asked me to come and talk to you about something before you see him. Would you please mind coming with me?” So, you blame it back to the doctor. So I mean it has to be, because the relationship is that GP relationship, so if we’re integrating other people into that relationship, it needs to be at the call of the patient or the doctor. PCMH 6, Practice Principal  *[name of GP] will actually walk them to my door and introduce myself directly and then I guess they see us working together as a team and therefore they accept me as part of the team that actually looks after them. So it actually improves the relationship between me and the patient and therefore they listen to me a little bit better and they are more happy to come back for reviews. PCMH 5, Dietician  *for example, spirometries can be done prior to the doctor seeing them. Care plan reviews can be started prior to the doctor seeing them and it makes the whole thing a lot easier. PCMH 8, Practice Principal  *I think our nurses are very valuable to us and then that’s been one of the things, and certainly our allied health care workers have been very valuable to us in terms of how we operate and how we run the centre ICP 3, Practice Principal  *of course the nurses, the practice nurses they are so valuable…and these registered nurses form a very integral part of our team. PCMH 2, Practice Principal B  *we have got a practice nurse now…so that has made a big improvement in the practice because when it comes to diabetic patients she helps us a lot. ICP 5, Practice Principal  *our nurses…take a lot of responsibility in tracking patient care over time and organising for patients to turn up on time for an annual health assessment to get their diabetes cycle of care complete, and to front for a care plan if they haven't got one and they've got complex care needs. Non-PCMH 9, Practice Principal  *I am the first here, but she [practice nurse] does half of my work. Non-PCMH 8, Practice Principal  *the medical management will be by the GP, but it will be more care management through the nurse. PCMH 7, Practice Manager  *****I think the biggest problem that needs to be overcome is the attitude of GPs, that they don’t like other people intruding into their patient care. It’s that old fashioned view of ownership and they want to feel like it’s their patient…I think it’s a big stumbling block PCMH 3, Practice Manager  *Everyone has to know the purpose of what their role is and what the bigger picture is PCMH 7, GP contractor  *I would like someone to give me a list and say, “Well, these will be all your duties.”…I don’t actually know what my role will be. What will I be expected to do? Other things that I’m not currently doing? PCMH 1, Practice Nurse |
| **Working at top of licence**   - *Team-based care using staff skills more efficiently* - *Team-based care allowing GPs to work at the top of their scope* - *Nurses working at the top of their scope* - *Aspiring to these changes in Non-PCMH practices* - *Some GP attitudes are a barrier to nurses working at top of licence* | *****there have been times where a consult with the patient where if it is a 20 minute consult, it’s 15 minutes with the nurse and 5 minutes with the doctor and the patient is very satisfied, it saves us time and it gets the nurse’s involve[d]. PCMH 2, Practice Principal B  *[Practice Principal] just does what he can do, so he’s operating at the top of his scope, rather than you know, weighing and measuring people. PCMH 3, Practice Manager  *all the nurses are always involved actively for the last few years. They collect their data from the patients and they contribute some clinical data for example, the diabetes they do the full check and they do the normal measurements and things like that and they always alert us exactly what should be done. PCMH 2, Practice Principal A  *I’m hoping it will free up doctor’s time to do more of the things that we do rather than some of the mundane things that we do. Non-PCMH 7, Practice Principal  *at the moment the practice nurse doesn’t do as much as we’d like probably in terms of checking everyone’s blood pressures or height and weight, but I guess if this [PCMH] comes on board then we can say to her, “Look, this is all part and parcel of the chronic disease management to do this,”…she’ll actually do more of what she would normally do. Non-PCMH 6, Practice Principal  *they don’t trust that their nurse can actually operate within the scope of their licence and do much more. They have this mindset that nurses do dressings and immunisations and that’s it. PCMH 3, Practice Manager  *I don't call them practice nurses because I find that if they are nurses and they're called practice nurses, they think they are de-facto practice managers in addition to their nursing role and I believe that there has to be a certain level of hierarchy Non-PCMH 10, Practice Principal |
| **Communication**   - *Time is required for regular team meetings* - *Clear communication helps in understanding purpose* - *Staff discussing patients’ needs together valued also in Non-PCMH practices* - *Staff sharing and learning together as part of culture including in Non-PCMH practices* - *GP isolation a barrier to communication and effective change* | *every Thursday we’ve got a meeting where the nurses and the doctors all get together, we discuss cases and all that. PCMH 2, Practice Principal B  *There has to be that protected meeting time…there has to be the time where people can actually communicate with each other and mostly for us it's been face-to-face communication but we have also done it as video conferencing as well when not everyone has been able to be here. PCMH 5, Practice Principal  *I feel that the meeting is very important because everyone on the team starts to understand why we're doing things PCMH 5, Dietician  *There should be very good lines of communication, so I think that’s something that’s integral. You need very good, clear communication and with purpose. PCMH 7, GP contractor  *Lots and lots of chats going on in corridors and people saying, “what about [name], did you see him the other day”?…there's no question that there's lots of that stuff happening where at least two people stand together and discuss a patient. Non-PCMH 9, Practice Principal  *you need that encouragement and ideas and sharing and we’re completely open to sharing whatever we do PCMH 4 Practice Principal  *we have principals in the practice who are all GP supervisors and we also get medical students in, so I think we’ve got a culture in the practice that’s a real sort of educational type culture, or a learning culture where we all try and learn from each other and so from that I guess we just sort of extend that into our nursing staff, allied health staff as well Non-PCMH 6, Practice Principal  *Often as GPs, we’re very isolated working, focusing on what we’re doing and if you don’t have that time to reflect or communicate and interact and actually talk about the issues and the problems or things that need changing, then changes don’t happen… You basically just keep doing the same thing. Former PCMH 1, GP Contractor  *unless there’s a major thing that affects them in terms of their clinical decision-making then they don’t necessarily want to get involved…they just prefer to come, see the patients, look after the patients and not be involved in that process. ICP 3, Practice Principal  *in their little silos basically running their own little mini shows everywhere…good doctors get their head down, bum up, just work, work, work, and “I couldn’t possibly have time to, how do I do that”, when they’re working so hard, just not realising that they're on a bit of a treadmill PCMH 4, Practice Principal |
| Staffing implications | |
| **Right staff**   - *Right staff have a common vision and purpose* - *Right staff are committed* - *Right staff are focused on patient outcomes not on throughput – noted also in Non-PCMH practices* - *Recruiting like-minded staff* - *Challenges in finding the right staff* - *Ageing workforce a challenge noted especially by non-PCMH practices* - *Recruiting staff is time consuming* - *Recruitment of allied health professionals and practices nurses is a challenge especially in lower capacity Non-PCMH practices* - *Increased hours needed for additional PCMH tasks* - *Need for well-trained nursing staff* - *Challenges in keeping a stable team* - *Loss of staff due to PCMH transition* - *More lucrative alternatives make it difficult to retain staff* | *it comes down to the people. You've got to have the right people on the team and having the vision, so making sure that everyone on the team actually has a common vision or purpose. PCMH 5, Practice Principal  *Either as registrars or as contractors, we want people with the same vision that they want to do the absolute best by the patient. PCMH 4, Practice Manager  *the main thing is I think the enthusiasm of the team, we always are a team based practise and every member of the team is committed. PCMH 2, Practice Principal B  *All the staff members are the sort of people who are interested in patient outcomes both the office staff and the nursing staff. So quality care as opposed to throughput and stuff like that. Non-PCMH 9, Practice Principal  *everyone who joins, joins at interview knowing that this is what’s here and this is what we expect and if you want to be a part of it, come, if you don’t we’re not for you…We’re very fortunate that we’ve, over the last year, managed to draw to us some more senior doctors who are pretty like-minded PCMH 4 Practice Principal  *you need to have all the stars aligned, have people with the right attitudes and the right skillsets coming in to interview and it is really hard. It’s hard to get good applicants. PCMH 3, Practice Manager  *the new generation doctors are reluctant to come to the west. I say that 70% of our doctors are over 50, probably a lot of them over 60 really and the challenges in the next few years when they retire, or cutting down their hours and we will be unable to recruit new general practitioners because they don’t come out this way. Recruitment is almost next to impossible Non-PCMH 2, Practice Principal  *we don’t have a dietician, we don’t have a psychologist and we need to look for some allied health professionals to come and work here, which takes a lot of my time too. ICP 5, Practice Principal  *it’s not easy to get a nurse for the practice. They used to come and they didn’t have enough work, and they were working part-time, and they wanted a full-time job. Former PCMH 2, Practice Principal  *As a solo practice, it’s very difficult for the doctor to employ the practice nurse and bring allied health in the same practice. Non-PCMH 8, Practice Principal  *I used to get dietician to come to the surgery and a psychologist, they don’t come anymore now because…I don’t get enough patients for them to see. Non-PCMH 8, Practice Principal  *our front desk staff are already doing maximum with their time. There's no free time for them, so we have to create more time for them to do these extra roles PCMH 5,GP Contractor  *your extra hours are also now incorporating 50 other things besides what you’re supposed to do. PCMH 1, Practice Nurse  *I come in an hour earlier two days a week and [name of staff] comes in an hour early two days a week and do the database thing. PCMH 1, Practice Manager  *when the nurses are more involved in the care plans and more involved with the health assessments, you need additional nursing hours…you just need extra nursing staff to be able to do the team-based care. PCMH 4, Practice Manager  *I just wish that if we had more support in terms of nurses being more qualified, we would be able to provide more care, better care Non-PCMH 10, Practice Principal  *my personal experience is the new generation of nurses who graduated from university know literally very, very little of general practice or even hospital...I have to teach them from ground zero up and it’s that bad Non-PCMH 2, Practice Principal  *we have also had team disruptions over the last few years as well…we've had a core team which has been stable but we've had others who have not been. So we need to stabilise. PCMH 5, Practice Principal  *every transformation talk that I’ve been to, whether it be a conference or a leadership course, they always say that when you start on this journey be prepared to lose about 30 percent of your staff. We’re up to 25 percent. PCMH 6, Practice Principal  *[GP registrars] usually get a better option, better offer. It’s a totally different type, but how on earth can you compete? So it’s very hard to retain PCMH 1, Practice Principal  *everyone is paid the minimum award rate and so hospital nursing becomes extremely attractive…the previous practice nurse got a job in interventional radiology…so I brought in a new nurse. She’s on $25.40 an hour and she’ll be gone in May…the hospital hourly rate plus the shift loadings and all of those things are extremely attractive and you’re very hard pressed to get a nurse who genuinely wants to stay in general practice nursing. PCMH 3, Practice Manager |
| **New staffing models**   - *Cultural shift required* - *New staffing model involves building teams and pods* - *New staffing model involves a panel of patients* - *Change in clinical workflows needed to support change in staff structure* - *Change to a more nurse-driven model of care* - *Increasing the nurse-doctor ratio* - *Funding for set quota of nurses a barrier to team-based care* - *New model explained to patient* | *the first change is culture and a shift in mentality of the staff from “my patient” to “our patient” PCMH 6, Practice Principal  *****it’s a paradigm shift when they start thinking about having a team look after the patient. PCMH 3, Practice Manager  *The pod is basically a team of people which consists of usually one senior doctor, one junior doctor and admin staff to basically do this proactive care for patient and then the team will have a panel of patients…so the pod is simply this structure of the whole group of staff with a panel of patients. PCMH 3 Practice Principal  *this is our plan, to divvy us up into probably two main groups but have each patient part of a smaller team so there will be the larger pod and then people who have a principal doctor, a secondary doctor, try and get that right with our hours, a registered nurse and an admin…and have [name of Practice Principal] and I as the senior GP, but not necessarily the patients’ primary GP and have each patient’s primary GP become a primary and a secondary, so instead of the patient just seeing any old person in the practice, when their primary person isn’t there, to have a secondary person so that they’ve got that person to be their go to. PCMH 4 Practice Principal  *we need to look at it operationally and then accordingly changing our staff structure to suit that. For example, it can’t be done by just a treatment from a nurse as a side thing… I’m looking at the clinical work flow that will need to change. How do we sort of get all that work flows happening effectively? PCMH 7, Practice Manager  *If there’s not enough doctors I think the PCMH model is more and more important to use, the allied health or  the nurses here to help you to do your work instead of you spending fifteen, you can spend five with the patient. PCMH 8, Practice Principal  *Because PCMH, I think in our views is that we’ll have to be strongly led by a nurse. So, the nurse will be the Care Manager, will be the Care Coordinator, that’s when it’s going to be successful you know. Doctors will need to have the input obviously, but it will be driven mainly by the nurse. PCMH 7, Practice Manager  *if it is a 20 minute consult, it’s 15 minutes with the nurse and 5 minutes with the doctor…it saves us time and it gets the nurse’s involve[d]. PCMH 2, Practice Principal B  *[name of nurse] pulls patients out of the waiting room now, does their height, their weight, their blood pressure, finds out what she needs, care plans…all that first before they actually see the doctor, prepares the things and gets him to sign and stuff like that. PCMH 1, Practice Manager  *our goal I believe is to increase the number of nurses to balance the doctors. PCMH 4, Practice Principal  *now we’ve got a ratio of one nurse to three doctors or even two doctors at certain times. Prior, we might have only had one, but we’ve upped the nurses at our expense…with this model it’s maybe a few doctors controlling X number of nurses. PCMH 8, Practice Principal.  *So at the moment the way it works we’ll only be able to have five full-time equivalents [nurses] pretty much and that may not be enough because I see so much value in having them sharing the care. PCMH 4, Practice Principal  *the medical assistant or the nurse explained to the patient the concept and then we got the patient to sign, so that they registered to be part of that team. So, explaining to them that what the advantages were and getting a commitment from them. PCMH 3, Practice Manager |
| **Co-location and space**   - *Working together as a team requires space* - *Non-PCMH Practices note a lack of space to be a barrier to PCMH model* - *Expanding space with technology* - *Additions to existing structures offer more space* - *Space needs to be considered carefully in the planning* | *It's just a space factor. We don't have a lot of space to do things. So sometimes that actually limits what we can do in terms of group sessions and things like that. PCMH 5, GP Contractor  *because it gets so hectic [staff name] can come and help, but then we don’t have a spare room for her. PCMH 1, Practice Manager  *we’re limited a little bit by space…we’ve only got four consulting rooms, so we’re not a huge, huge practice. Non-PCMH 6, Practice Principal  *we’d like more space to do some of these things…if you’re going to have case conferences and those types of things you’d need the space to have that somewhere. Non-PCMH 7, Practice Principal  *we've only got this room here so if we have a practice nurse, the allied health professional has to come when the practice nurse isn’t here, so there's a limited amount of time…we can’t add on, so we cannot give you allied health professional rooms…there’s only two consulting rooms so only two doctors can work at any one time. Non-PCMH 4, Practice Principal  *at the moment as far as we can see there’s no room for expansion. Non-PCMH 7, Practice Principal  *they sat in another room and they video-conferenced in from a different room into this room so that we were able to expand the room. PCMH 5, Practice Principal  *We haven’t got any rooms for any of the allied health personnel so I’m just buying a property next door where there will be larger space. There’ll be five or six rooms…this is what prompted me to do it, because we haven’t got space. ICP 5, Practice Principal  *modifications can be made on level two rooms. It’s partial at the back, so without moving the premises there’s still room to expand the practice. ICP 1, Practice Principal  *We actually built this building before we fully understood where we were heading and having kind of seen a vision I suppose of what’s coming we can see now that we made the rooms too big. For instance, we should have had a couple of smaller rooms and then some interview areas so that we could do little team sessions PCMH 4, Practice Principal  *the treatment room has a computer as well as the nurses’ rooms. Now, we’re finding now, that even though we thought this was so well resourced we need another workbench and another computer in there because there’s too much stuff going on and we just need more IT. PCMH 4, Practice Manager |
| Data driven care | |
| **Quality Improvement**   - *Having time for data* - *Clinical audit tool enables data driven care* - *Manual extraction of data is time wasting* - *Need for well coded Electronic Medical Record* - *Importance of collecting comprehensive high quality patient data* - *Using data to see where quality improvement is needed* - *Using PEN CAT to plan and prioritise patient care including reminders and recalls* - *Using PEN CAT data to track population health* - *Using data comparisons between practices to improve care* - *Staged approach to improving care based on data driven quality improvement* - *Using PDSA cycles and KPIs to encourage quality improvement* - *Opportunities for Non-PCMH practices to be involved with quality improvement* | *you must have the time for data PCMH 5, Practice Principal  *having a clinical audit tool is an absolute must and enabler. Whether it's PEN, whether it's a different tool, but some way of auditing PCMH 5, Practice Principal  *So based on what we have currently, having other programs like the PEN CAT data because everything is coded where you can search obviously has helped immensely. You can imagine if everything was still on paper it would be impossible to do any of these things at all. PCMH 3, Practice Principal  *this is another level of using PEN CAT to data extract…we are trying to identify as part of a submission to the department, like how many patients do we have with cardiovascular and diabetes. PCMH 7, Practice Manager  *we’re tracking our PEN CAT. [Name of nurse] does all the PEN CAT and at the end of the month I pull out how many diabetes stats we’ve done, how many care plans... PCMH 1, Practice Manager  *a lot of that time is manually extracting the data, cleaning it up because the extractor tool does so much but then you've got to go through and check is it all correct, what's missing…and ways of presenting the data manually is taking time as well. So that's definitely hindering the process. PCMH 5, GP Contractor  *Having a well coded and comprehensive EMR is very important. So that's the first - that's the basic steps. The next step is ensuring that everyone uses the EMR, everyone reads and writes the EMR. PCMH 5, Practice Principal  *The team of doctors we have now is really into correct medical record taking and correct coding and correct, if you like, taking out – so we’ve cleaned out that dramatically over the past few months. PCMH 1, Practice Principal  *Our first goal is to improve the quality of the data…the fact that you’re going into someone’s file, reviewing their history, cleaning up the past history and cleaning up all that stuff that is in the file that isn’t relevant, means that you’re doing something useful PCMH 6, Practice Principal  *we’ve been very pedantic about collection of data, every single bit has to be there…we have put steps in place from the front of desk to the nurses to the doctors encouraging them to collect as much data as possible. PCMH 2, Practice Principal B  *we’re trying to get better data, so we’re looking at doctor’s data or individual data about management and things like diabetes and we’ll have meetings to discuss that and hopefully we’re looking after those metrics a bit better. PCMH 6, GP Contractor  *at first it was just improving data quality and then once we've got good data actually analysing it and seeing where we need to improve and then focusing on those areas. PCMH 5, GP Contractor  *[PEN CAT] helps with individual patient management in terms of planning the patient's care for example, diabetic reviews and care plan reviews and blood tests and preventative care, like FOB tests and PAP smears. PCMH 5, GP Contractor  *it's very useful for me to be able to access it [PEN CAT] and read and see results. It's just helping in the way of prioritising what I should do for a patient. PCMH 5, Practice Principal  *utilising the computer systems that we do have to improve our planned care, that's been really good for example something as simple as the recall list, a very simple function PCMH 5, GP Contractor  *we go through the PEN CAT and see what has not been done and so on, so we are trying to complete the gaps of our patients, the clinical management of chronic diseases. PCMH 2, Practice Principal A  *PEN has been fabulous because you can create an Excel spreadsheet of this doctor’s patients who are diabetic, this doctor’s patients who have heart disease and then from there you can look at the targets. PCMH 3, Practice Manager  *the PEN CAT tool that WentWest provide for us is great for that because you can look at all of your disease states and all of your targets and it’s not just my patients are doing well, it’s X per cent of my patients have achieved an HBA1C that is below seven, or Y amount of my patients have their blood pressure under the specified target. PCMH 4, Practice Manager  *The opportunities that we’ve had through WentWest to have data comparisons in the area has been helpful so we can see how we’re going…I need to know how I compare with other doctors. I want to know who’s doing better than me at certain things so I can pick their brain on how to do better PCMH 4, Practice Principal  *it’s good to get that feedback about how your data compares to other practices…and how your data compares over time. Former PCMH 1, GP Contractor  *we discuss about what is the data that we need to check for this chronic disease. So we start with diabetes and move onto asthma and move on to – so it’s one at a time to decide on the thing we need to check on. PCMH 3 Practice Principal  *probably maybe a year ago, if you asked us how many patients with blah have you got, we wouldn’t know. But now we can tell you and not only that, but we know how many of those patients have or haven’t had something done to them. That’s the difference - knowledge driven by data is changing. PCMH 6, Practice Principal  *with those PDSA cycles that we’ve done, we can then sit back, consolidate, look what is it we need to do? How will we do it? PCMH 7, Practice Manager  *we were involved with the Improvement Foundation and that was basically looking at improving chronic kidney disease within our practice…there was a whole bunch of things called PDSA Cycles Non-PCMH 7, Practice Principal  *for full health assessment, annual health assessments and care plans and other associated activities for people with chronic and complex conditions, and the immunisation side of it we use the computer to help us track all that. Non-PCMH 9, Practice Principal |
| Information technologies | |
| **Use of computer software systems**   - *Need for one integrated electronic system across general practice and hospitals* - *IT programs not working well is an obstacle* - *Problems with Linked-EHR* - *IT issues causing GPs to stop using software* - *Time consuming manual input required* - *Linked-EHR not known by allied health* - *My Health Record difficult to use* - *Lack of appropriate IT programs are an obstacle* | *we have to just really get a system, one system, or a system that will talk to other systems so that it’s all, but it needs to be real time. PCMH 4, Practice Principal  *I should be able to see the patient’s documentation when they’re in hospital. I should see what the specialist thought of them on their round that day. PCMH 4 Practice Principal  *IT is I think probably one of the major obstacles PCMH 2, Practice Principal A  *There is no doubt that one of our major handicaps is our software. PCMH 1, Practice Principal  *I’m still having problems with my uploading - best practice. They’ve been here countless times, trying to fix that up. PCMH 8, Practice Principal  *the E-linked health record…sometimes it doesn’t always work so good, there’s always some kind of a problem there PCMH 2, Practice Principal B  *my personal opinion is that they shouldn’t have launched it [Linked-EHR]. They need to launch an upgrade of it as soon as possible, because it’s not a nice system. Even the appointment book has issues. So the Linked-EHR is nice in its concept, but really difficult in the implementation. PCMH 4, Practice Manager  *In terms of what are not enablers or what has held things back, unfortunately Linked-EHR has not been a good thing. It has all the best intentions but the execution has been clunky and it hasn't progressed as it should have PCMH 5, Practice Principal  *we couldn’t use the internet with it, so it’s like if I’m trying to do the Linked-EHR care plan, I can’t open Top Bar and do anything with that because the internet is open PCMH 8, Practice Nurse  *there are lots of GPs in our last meeting for example they stopped using the service because it’s a headache, so I stopped and once I stopped and I don’t use it I forget it. PCMH 1, Practice Principal  *the toolbar does not talk to the Linked-EHR and they have to physically put the stuff...The software should be all integrated together. PCMH 1, Practice Principal  *things aren’t loaded up automatically, like the clinical metrics. If someone wants that, the current one uploaded, then you need to ring and tell me if you want it, because it doesn’t automatically come from best practice - it’s got to be manually done…so that’s time consuming PCMH 1, Practice Nurse  *What happens with the Linked-EHR is you have to do a whole other care plan for the patient. You can’t take the care plan that’s already in best practice and put it in there. So it’s double work for somebody and it’s either double work for the doctor or double work for the nurse. PCMH 4, Practice Manager  *It’s actually not duplicating, it’s triplicating the work that she’s doing and then they [GPs] just refuse to use it [software]. It needs all to be a little bit more simpler. PCMH 1, Practice Manager  *If I’ve sent them [allied health] a copy of the Linked-EHR they’re going to look at it and think what the hell is this? Because I mean just the format and everything PCMH 1, Practice Nurse  *The My Health Record – sometimes it’s easy to do, sometimes it’s not. We were having – with the new version of Lava that we have here, for best practice, every time somebody went to do a My Health record for a patient, the system would crash. You know doctors will try and do a shared health summary and then it would freeze and then they’d lose their notes for the session. PCMH 4, Practice Manager  *My Health Record is even worse. It's even more clunky and even more useless to us. So if we didn't have to upload 0.5 per cent of our standard whole patient equivalent numbers of shared health summaries per quarter we wouldn't be doing it at all. It's just another thing which is wasting time. PCMH 5, Practice Principal  *I guess the setback would be what we mentioned earlier about not having the appropriate software. PCMH 3, Practice Principal  *lack of appropriate IT…we’re trying to write formulas on the excel spreadsheet to indicate ok well if this is a certain number or under a certain number then this will become yes or no…and then how to actually do the formula to cut off and they have a main excel sheet that links to all these other sub-sheets to see which patient is required to actually be contacted or not, and that’s what we find has the biggest road block to try and get that running. PCMH 3, Practice Principal |
| Communication with external stakeholders | |
| **Use of electronic communication**   - *Receiving hospital discharge summaries electronically* - *Hospital discharge summaries not user-friendly and not consistently received* - *Integrated care plans not easily accessible in the hospital setting* - *Integrated care program not known by other health providers* - *Lack of communication between General Practice and external providers* | *Doing the patient electronic health record, that’s not too bad. We get a lot of discharge summaries electronically now, which is good. PCMH 8, Practice Principal  *we’re getting lots of referrals from all the hospitals really good now. They come up very promptly, almost immediately. We get them electronically so that actually works really well PCMH 6, GP Contractor  *we still have to call especially at the hospitals. You never get anything from the hospitals, they don’t communicate and it’s got to be also in a format where it’s easy to read. You can’t go through a ten-page discharge summary. You’ve just got to see the abnormalities in red, or something like that and a good conclusion. PCMH 8, Practice Principal  *Because he signed himself out [of hospital] I was not allowed to have access, there’s no discharge summary, I’m not allowed to have copies of the notes. PCMH 4, Practice Principal  *we’ve had patients who have been discharged and you know how they say, “send it electronically”. We try and find the discharge summaries electronically – never there. PCMH 1, Practice Nurse  *the hospitals end of it, they’ve got to go through separate screens to actually click to find [the care plan]. So they’re in ED, they’re too busy to go through seven screens. It’s easier just to make a phone call to their local GP. PCMH 1, Practice Manager  *someone rang up about integrated care, or they wanted some information about a patient. [Name of Practice Manager] said to them, “They’re in the integrated care program. All the information will be on the care plan.” Didn’t even know what integrated care was, didn’t know. PCMH 1, Practice nurse  *in the public system they’re not co-operating, they’re not communicating well in a timely fashion… you go around in circles. Non-PCMH 1, Practice Principal  *we’re forever chasing pathology results for specialist’s reports or radiology results to get downloaded into our system Non-PCMH 7, Practice Principal |
| Fee for Service | |
| **Constraints**   - *Medicare model not good fit for PCMH model of care* - *Medicare encouraging throughput not quality practice – concern shared by PCMH and non PCMH practices* - *Value of care plans perceived in terms of financial remuneration* - *Health sector money not spent wisely* - *Medicare freeze is a constraint* - *Costs not reimbursed under Medicare model* - *Need for re-imbursement for different care provision e.g. phone consultations* - *Costs of running the practice a challenge* - *Patients missing appointments a financial burden* - *Perceived undervaluing of general practice by patients* - *Bulk billing necessary due to patients from low socio-economic backgrounds* - *Patients unable to afford specialist costs* - *Additional burden for GPs taking on specialist care* - *Specialist care in general practices seen as an advantage* - *Funding needed for practice nurses* - *Change in funding needed so that PCMH strategies can be supported* - *Support and training required to improve billing* - *Challenges of dual system – Medicare and PCMH* | *at the moment it's a billing system, fee for service system, so we're working within the confines of that system…it does affect the way you would do things if you're working with the medical home in mind. A lot of things that you would do from a medical home perspective don't always involve face-to-face care, so case conferencing with your team about the patients for example, morning huddles, afternoon huddles where we're talking about planning the care with the people here every day, like the nurse and the front desk…that all doesn't get involved with any kind of payment at this stage and then any phone calls, follow-up, all that kind of stuff it's not part of Medicare's payment model. PCMH 5, GP Contractor  *there is a lot of time getting spent on meetings, on discussions…at the moment that has been funded out of our pockets because everyone is donating their time basically. PCMH 5, Practice Principal  *in the current fee for service model the only way you can generate income for the practice is to see patients and then that really leaves you stuck on the same road PCMH 5, GP Contractor  *It’s very disturbing that the people who choose to see someone for six minutes, ten times an hour make twice as much as we do in spending time with someone who needs us for an hour and I think that unfortunately has been perpetuated long enough that there are those who can’t see any other way PCMH 4, Practice Principal  *a lot of doctors just want to go into big bulk billing practices and have their ten minutes with patients and go home and offload to the allied health…I can't even get a doctor to come and have any interest in what I do, because I spend half an hour with a patient. Non-PCMH 5, Practice Principal  *None of that's reimbursed. It’s all taken for granted…it’s because we are only paid per face. We are paid the minute the person sits there and as soon as it stops. Non-PCMH 4, Practice Principal  *One care plan is what $250, to see a consultant is less than $40. You can see six patients instead of a care plan. If a doctor is trying to make money, rip off the system would you do a care plan or would you like to see six patients? Non-PCMH 2, Practice Principal  *getting paid for GP management plans and all that sort of stuff, which is just rubbish…because people just churn them out. So if you go to a medical centre, they’ll churn one out to get the money whereas they’re not actually looking after the patient. Non-PCMH 4, Practice Principal  *The amount of care plan done by a GP and wasting government money can easily pay specialists in a multi-disciplinary team to look after those chronic disease patients with a much better outcome Non-PCMH 2, Practice Principal  *I have a feeling that we’re spending too much money but we’re not spending it wisely…Every time we put money actually it just basically is so many holes it gets spent the wrong way, there is so many bureaucracy, there is so many paperwork has to be filled and so many nursing staff here and there, administration is horrendous everywhere but at the end of the day we’re spending more money Non-PCMH 1, Practice Principal  *We are a small practice, we bulk bill, we only book four patients an hour. We are doing extremely well, but we can do a lot more but not with the current restraints, especially with Medicare freeze. PCMH 1, Practice Principal  *our biggest challenge at the moment is the cost of running the practice…because ultimately Medicare hasn’t changed in over 10 years enough…at the same time our costs keep going up every year. ICP 3, Practice Principal  *Medicare used to have a billing item number for dressings which they’ve removed and so since that’s been removed we’ve just kept up doing what we’re doing without, so that we bear the costs. ICP 3, Practice Principal  *There are lots of stuff I can sort on the phone, but when I do it on the phone I don’t get paid, which is really stupid, but if you call and say, “I want my contraceptive pill,” okay, if everything is good, why do I bring you here? And I really think that if you have a chemist next door to where you live I should be able to send it electronically there, but then I don’t get paid PCMH 1, Practice Principal  *everything that comes out of his business comes out of his pocket…when [name of staff] is fully trained then she will have to get a pay rise and that’s got to come out of him and then if I moved away from the desk then he has to pay someone else to sit at the desk. PCMH 1, Practice Nurse  *We don’t have money aside to, say for example, the girl who is going to do a medical assistant, I want to take her out of the front desk. It’s impossible at the moment. Money-wise it’s quite impossible. PCMH 1, Practice Principal  *one of the big difficulty with us is how we bill and basically sometimes people don’t turn up and we can’t bill for that either and so you lose income…the rate of missing appointment is about 20%. ICP 3, Practice Principal  *we have every day eight people not turning up and that causes lots of financial burden. PCMH 1, Practice Principal  *How do we get patients to care about their health more? I don’t think it was a bad idea to charge a co-payment. Patients would come in and they would have to pay a small amount but that means they value the service…at the moment it’s just whatever you want, whenever you want. PCMH 8, Practice Principal  *Anything that is free is underestimated, taken for granted. The worth of it becomes meaningless. So as soon as they made Medicare free for everyone, it becomes a problem. Non-PCMH 4, Practice Principal  *at the moment it’s just free for all, people are just going all over the place. PCMH 7, GP contractor  *patients have had so many reasons to disrespect doctors over the years because of what’s happened and really since the 70s and when Medicare started it’s been this gradual change that’s encouraged faster throughput because of the billing purposes, and less self-respect on the part of some doctors and respect for doctors on the part of patients PCMH 4, Practice Principal  *But if you don’t bulk bill, you can’t survive here…nobody will come to you if you charge them. Former PCMH 2, Practice Principal  *I think at the moment the payment system is one of the biggest problems that we have because we’re bulk billing. Unfortunately in this area, we realise how difficult it is for the patients and how much it costs for them and so we haven’t been able to get away from the bulk billing ICP 3, Practice Principal  *a challenge from a patient point of view is being able to see the specialist that they need to see and we end up doing a lot of the specialist care which we haven’t got the specialist skills that are needed sometimes, nor the equipment. ICP 3, Practice Principal  *Providing a practice with say the specialist that comes every three months so you know cardio, endocrine and respiratory would be the top three ones that patients are always nearly needing to be referred to or can’t afford because of the cost, particularly the elderly. PCMH 7, GP contractor  *We are not big enough to fund the nurse without the funding…they squeeze, squeeze, squeeze that too by the way, so as soon as that funding stops the nurse has to go. Non-PCMH 4, Practice Principal  *if there was a change in funding or a change in resources that would allow - even more grants and things to trial new things… then you can provide evidence that things are working and what is beneficial PCMH 5, GP Contractor  *So funding for a data manager, funding for meeting times, funding for case conferencing times, all of those things where you can plan the care for the patients, plan the care for your population PCMH 5, GP Contractor  *when you are working it does not cross [your mind] that this qualifies for this, this qualifies for that and that’s why we lose lots of money compared to more sophisticated practices where people are sitting on a desk somewhere and bringing up that with this patient we can bill on this, this patient we can bill on that, so you increase the billing. PCMH 1, Practice Principal  *how do you do Medicare and how do you charge the other ones? Now this isn’t a patient centred, this is a Medicare, this is this one or he’s already had his allocated, who pays for that consultation? PCMH 1, Practice Manager |
| Uncertainty regarding PCMH and HCH models | |
| **Funding uncertainties**   - *Uncertainty about funding models* - *HCH still a type of fee for service* - *PCMH strategies not funded in HCH model* - *Inadequate funding for chronic disease patients* - *Inadequate funding barrier to motivation* - *Challenges of working two payment systems and who gets paid* - *Lack of funding for non-GP staff* - *Concerns that lump sum payment might not encourage quality care* - *Administrative burden* - *HCH could be financially damaging* - *HCH could be damaging for PCMH model* | *our issues and concerns are mainly about the unknown-ness of it [HCH model]…You've got people like the prior college president saying you really need about a hundred thousand dollars of seed funding to make this work properly, and of course we get ten thousand dollars’ worth of seed funding. Non-PCMH 9, Practice Principal  *we have some reservations about that funding model [HCH]. We are still not sure how it’s going to work. PCMH 2, Practice Principal A  *I can only see it [PCMH model] as a mechanism of the government to reduce funding to general practice. My cynical view is this is just another way of Medicare, of the government trying to reduce costs. Non-PCMH 5, Practice Principal  *The only drawback is we have to look at what the financial issues are and the funding model that’s there and whether that sort of stacks up to what we currently get paid. Non-PCMH 6, Practice Principal  *Definitely, the cost is going to go high if I have more health professionals working here. So I don’t know how the funding is going to help us or if there is anything. I’m not sure. ICP 5, Practice Principal  *If the chronic disease management item numbers, health assessments and care planning go then there’ll be no money. PCMH 4, Practice Principal  *if the funding attached to it, which as it currently is, is based on an average Medicare outlay for an average patient, it's really still just another form of fee for service except that it's fee for service paid as a block funding. PCMH 5, Practice Principal  *the problem is that the quantum of funding still doesn't match because the existing funding doesn't take into account the unpaid time, the meeting time, the data time, the time for reflection and education and the funding for health care homes basically means that that time has still not been taken into account. PCMH 5, Practice Principal  *the funding they actually allocate for chronic disease patients is grossly inadequate so if we actually sign up for that we have less resource to try to do what we are trying to achieve. PCMH 3, Practice Principal  *In terms of the current one that's coming out, the Federal Government Health Care Homes, it's a bit tricky, that one. It's something that is good in principle, in theory but the numbers don't really add up in terms of what they've thought patients would need and what's available to carry out the care for those patients…for those top tier patients with multiple co-morbidities we've kind of had a look at it roughly and we think that that funding is not adequate to do the things that we've already been doing for them. PCMH 5, GP Contractor  *if people with very complex care needs who see us a lot and who take a lot of our time up…if it were thrust upon us that those people should have a fixed annual fee which is lower or no more than the current fee, then it'll be an awful lot of work for no use at all. We won't feel at all motivated. Non-PCMH 9, Practice Principal  *the HCH model with the payment they actually currently have the lump sum only for the practice and for the practice to separate it and I think that makes it quite tricky and it excludes doctors from billing the patient subsequent visits. PCMH 3, Practice Principal  *there’s the ambiguity about if you get X amount of dollars for the practice then how on earth do you distribute that? PCMH 4, Practice Manager  *GPs, a lot work part-time, so say if I see a patient one time and somebody else sees a patient another time, then how we’d divide up that money at the end of the day? PCMH 7, GP contractor  *the first instinct for a contracting doctor is going to be if you have all staff doing all that then what, how do I earn money? PCMH 4, Practice Principal  *from our point of view yes it’s doable, our challenge is the funding for all the non-medical staff, so like a medical student assistant, there’s no funding for that, even the registered nurses once you’ve gone beyond five there’s no help with that. PCMH 4, Practice Principal  *there was going to be like a lump sum payment for practices and I think that was going to be a problem and a lot of GPs were reluctant because it would be just like a money grab at the end, like how many patients can we all sign up quickly so we can get that initial lump sum and then not do anything with them PCMH 7, GP contractor  *****It’s going to be more for us at the desk having to work it out PCMH 1, Practice Manager  *it may actually be a bigger problem because if, for example health care homes required more time to manage financially and more time to report on it then it actually could be a distraction. PCMH 5, Practice Principal  *it will basically put us backwards [HCH]…if we actually sign up for that we have less resource to try to do what we are trying to achieve. PCMH 3, Practice Principal  *we’ve just sort of done that leap of faith and gone “well, I hope financially we’re not disadvantaged”. It is really tricky because some of our patients when we tried to do sums look like we would actually be losing out because of the number of times that they were in. PCMH 4, Practice Manager  *we're not interested in operating in an unfunded way… if it's workable and we don't have ongoing battles going backwards money-wise then we'll get on with it. Non-PCMH 9, Practice Principal  *my personal thought is it might be damaging to the patient centred medical homes…in terms that it might actually make general practitioners think that they can't do it, they're not the right amount of resources to do the care that we would want to do for those patients…so that might be a damaging factor, that it might get more practices opting out of the medical home model. PCMH 5, GP Contractor |
| Early outcomes of a PCMH model | |
| Patients | |
| **Patient-centred care**   - *Putting the patient first* - *Patients as members of the team* - *Involving patients in decision-making and management of care* - *Educating patients about taking responsibility for their health* - *Patients being able to access and control their electronic health record* | *we always put the patient first and try to satisfy all their health needs. PCMH 2, Practice Principal B  *to create a type of general practice that it is comprehensive and it is affordable and it is pro-patient, like you put your patient as number one within realistic goals. PCMH 1, Practice Principal  *when you look at patient centredness, a very important element is how we engage with patients in terms of keeping them communicated. PCMH 7, Practice Manager  *we actually wrote as our very first philosophy of the practice mission statement that our patients would join us and become members of the team so that we see them as part of the whole thing. PCMH 4, Practice Principal  *My practice has always been it is your responsibility to look after your health, I’m here to help you and to assist you and to get you better but you need to put in your ounce of work…you need to take that responsibility ICP 3, Practice Principal  *we spend our whole time trying to educate patients to take responsibility for themselves, look after themselves Non-PCMH 4, Practice Principal  *patients actually being involved in their management…patients are listened to and what they want is taken into consideration and not directed by a guideline that’s got sets of tick boxes. Non-PCMH 5, Practice Principal  *more group sessions, more shared medical appointments, things like that where the patients get more involved in their care and helping them be part of their decision-making process PCMH 5, GP Contractor  *we use My Health Record saying that you can take your health record with you and a lot of tech savvy patients are quite happy seeing the electronic health record uploaded. PCMH 2, Practice Principal B  *it’s imperative that patients know what I put in my notes and can read, correct it if they want to…the patient’s at the core of it and has control of it and can see it PCMH 4, Practice Principal |
| **Patient Education**   - *Educating patients about valuing health care* - *Changing patient attitudes towards the health system* - *Educating patients about value over convenience* - *Increasing patient awareness of PCMH and what it provides* - *Increasing patient awareness of what is expected of them* - *Increasing patient awareness of benefits of registering* - *Importance of continuity of care for patients with chronic illness* - *Increasing patient awareness of available health care services* | *There should be more emphasis on patient education. I think over the years we have neglected teaching the community about health and people take for granted that if I don’t turn up so what…we need to change that PCMH 1, Practice Principal  *the integrated care nurse she makes a lot of bookings but a lot of the people don’t come and I think that is again the patient thinking what’s the value for me? What am I getting out of this? PCMH 7, GP Contractor  *what are patients going to see as the value of coming to one practice as opposed to the convenience of going to whatever practice is nearby at the time? PCMH 7, GP Contractor  *one of the things that is on our to do list, getting very high at the moment is to spend some more time documenting both on our website, Facebook and other opportunities in newsletters to talk about what to expect from a visit to the doctor, what your commitment needs to be if you’re going to get the best out of it, because people don’t seem to know what they can get from a consultation in a primary care setting and I think the concept of the PCMH is so far away from what most people would have experienced. PCMH 4, Practice Principal  *I think firstly would be definitely education of the greater community, say explaining in simple terms what does this mean to them. 1) you nominate your medical centre as your home. You will go there for all your needs and 2) that it will actually have the needs. PCMH 7, GP contractor  *Making sure that the patients are up to date with our services that we give and they know what we expect from them. Making sure that the patient knows that this is your practice, this is where you’re going to get your care and all your health needs will be done at this particular practice…we’ve been registering patients and making sure that they are responsible and they need to come and see their practice, their doctor PCMH 2, Practice Principal B  *I think to create a much greater general population understanding and cultural shift towards the fact that yes, this medical clinic is my medical centre, this is where I go to for all my needs. PCMH 7, GP Contractor  *Anyone who comes to us are told that it is as if you are registered with us, especially if you do have a chronic problem that needs regular maintenance. Then it is up to you to make sure that you come and see us on a regular basis, we’ll do our best to make sure your visit with us is as enjoyable as possible. But one of the prerequisites is you have to be with us on a regular basis…the patients as well know that if they’ve got a medical problem that is an ongoing thing they need to come and stick to one practice and one doctor. PCMH 2, Practice Principal B  *I don’t think they know of a lot of the avenues open to them…she [patient] was like what is a diabetes educator? She wasn’t aware of what everything meant and how I could help her, and I was like here is someone that will explain everything to you. So yeah, patient education to let them know, not even just integrated care but just what is available to them and why we see them and do what we do PCMH 8, Practice Nurse |
| **Holistic Care**   - *Cultural shift to Preventative care* - *Looking after patients comprehensively and holistically* - *Using software to proactively provide health care* - *Educating patients about the need for regular appointments* - *Patients not understanding about the need for holistic, preventative care* | *I’ve been in about five or six other practices before and I think the focus there is really point of care. Like when they come in they come in for a particular issue, deal with that issue and move on…the medical concept is we need to look at your overall health. PCMH 7, GP contractor  *this practice is more like caring, compassionate, personalised service, comprehensive. You don’t get rushed in and out in five minutes. ICP 1, Practice Principal  *prevention is obviously the major thing. So, rather than someone sitting there all day on chest pain, not wanting to come in, and then getting sent to hospital, they come in early and we catch it early PCMH 8, Practice Nurse  *we take great pride in making sure a patient’s immediate problem is dealt with, their preventative health is dealt with, seeing what happened in the last consultation is dealt with and also formalising that in a kind of reminder list for them which we have on our best practice set up for each patient. PCMH 8, Practice Principal  *I do really appreciate that they're trying to make things work for a broader population of people than for people who are sick. I think it’s got a place and I think it’s noble. Non-PCMH 5, Practice Principal  *slowly build the program so making sure all the reminder recalls go out…use the data to see what are the patients who are not coming in to actually try to engage them PCMH 3, Practice Principal  *The program should automatically run through and remind them or flag it a certain way, I think that would be the vision…Every patient will be taken care of proactively rather than reactively, only when they come in when they’re sick. PCMH 3, Practice Principal  *The vision is that every patient that has actually associated with us as us being their regular carer that we should be having what I call a clinical pathway setup for them so we know how to take care of them and all of these things built into the software where everything is proactively followed up. PCMH 3 Practice Principal  *you’re due for your three monthly check-up for blood tests and it has like a schedule for the year. So you’re mentally preparing the patient that you don’t just have to come here just when you’re sick. We need to schedule these in as you would your hairdresser or as a regular appointment. PCMH 7, GP Contractor  *they get almost offended when they’re asked about their general health issues and have you thought maybe you should do some bowel cancer screening…“why would you bother asking me that, I just came here for a cold” or “I just came here because I need a script for my blood pressure”, “you don’t have to tell me how to run my life” and “I don't really want to”. PCMH 4, Practice Principal  *when I’m seeing someone for an acute condition and I will see opportunistically that they have had a history of diabetes and I will ask them, “when was your last diabetes check?” And there’s always that reluctance going “oh no, things are fine”…they’re like, “well it’s not affecting me so I’m not going to put time into it”…they don’t see the value. PCMH 7, GP Contractor  *Even getting them in to come and get their blood pressure taken, oh my God, some of them think, “why am I having my blood pressure taken. What’s going on?” PCMH 1, Practice Nurse |
| **Continuity of care**   - *Providing good quality care by consistent carers helps facilitate continuity of care* - *Patient registration helps facilitate continuity of care* - *Using patient registration to help manage chronic care patients* - *Seeing multiple doctors is a barrier to continuity of care* | *we got a lot of patients involved in this program, the patient centred medical home and then we managed to follow through those patients more effectively than maybe five years before all this...and the patients are happy about that, the way we are doing it PCMH 2, Practice Principal A  *what we initially started was to try and obviously get a lot of continuity of care and get our patients to come back to the practice and I guess if we give a good quality of care then they’re more likely to come back…the majority of the time they’ll come back to us for whatever health needs that they have and that’s also got to do with the stability of the doctors in the practice as well. Non-PCMH 6, Practice Principal  *Well, I think the nature of our practice is that we have very long serving doctors and nurses and receptionists, and it's an old practice that everyone knows where it is in [name], and a group of [location] have chosen us and keep coming back…stable staff, stable patients Non-PCMH 9, Practice Principal  *if they come into the same practice and we’ve got all that information then it’s easier to get your head around what’s been happening with this patient…if we had them registered then that would make that a lot easier. Non-PCMH 7, Practice Principal  *management of chronic disease always works better if you’ve got patients who are committed to our practice and within the practice committed to a particular doctor, so we started doing registration of practice of patients long before the PCMH existed. PCMH 2, Practice Principal B  *if they [patients] see different doctors then things get lost and there’s no one cohesive place where all these plans are actually kept and followed up. PCMH 3 Practice Principal  *You still get patients with chronic disease who will still come to see us for certain things but will also see a second GP or a third GP for the same condition and to try and collate all that together and give them bits of information can be difficult. Non-PCMH 7, Practice Principal |
| **Accessible care**   - *Appointments made available for registered patients* - *Access to services in one location is convenient for patients* - *One location is particularly convenient for elderly patients* - *Multidisciplinary team care accessible to patients in one location* - *One location enables efficiency of patient care* - *Multi-disciplinary team helps reduce patient waiting time* | *we also explained to them [patients] that being part of that team meant that there would be appointment slots that were set aside for them. PCMH 3, Practice Manager  *to make sure that appointments are available with the team members that they need to see, with the doctors, with the nurses, allied health PCMH 5, GP Contractor  *obviously if you were designing your centre to be the home for that patient you need to be able to provide that service for them. So another drawcard for the patient could be, if you register for us you will be guaranteed to get an appointment in. PCMH 7, GP Contractor  *Before when we didn’t have X-rays here or physio here, it was “do I have to go to Blacktown to get my X-ray?” and “I’ve got to wait half an hour for you, to see you, have to drive to Blacktown, look for parking, get the X-ray, come back, wait again, look for parking” and it was disastrous. PCMH 8, Practice Principal  *certainly the patients find the convenience useful and they certainly like to come to one place rather than trips here and there given the difficulty with parking and things ICP 3, Practice Principal  *with the elderly patients I think that’s even more important because they use the one location and so it’s easier for them to come to the one location, particularly the ones who don’t have the family support. ICP 3, Practice Principal  *it’s like in one place they can come and see the nurse, talk to us and get some help from the physiotherapists, if they want to lose weight they can see the dietician, they can probably see an exercise physiologist all in one place, so they don’t have to travel from here to there, they get the service in one place. ICP 5, Practice Principal  *Patients said they’re happy because they get information [from pharmacist in General Practice], the more they know the better. Former PCMH 2, Practice Principal  *I can see the value of the other GPs, nurses and other allied health working together. A lot more things can be  done for the patient a bit more efficiently…if everybody’s on site ICP 1, Practice Principal  *in order to reduce the waiting time we get everybody else involved, so a patient does not need to wait so long because most of the care will be done by the nurse and the doctor has to come in and just do the basic stuff PCMH 2, Practice Principal B |
| **Adopting new approaches**   - *Patients benefit from engaging with new approaches* - *Patients at centre of team approach* - *Understanding patients from different cultural contexts* - *Providing a more comprehensive service with team-based approach* - *Using all members of the care team to get best health outcomes for patients* - *Multidisciplinary team involved in case conferencing and reviewing care plans* - *Enabling GPs to spend time on establishing management framework for patients* - *Patients not understanding team-based approach* - *Understanding and adopting new approaches is particularly challenging for elderly patients* | *a lot of patients with chronic medical conditions they are looking for more engagement in their care I think. So you will get some patients that will probably be the early adopters and they love the group work and they love coming into group sessions and doing things like shared medical appointments and education sessions and seeing the allied health team and the pharmacist more frequently. They really engage quickly. PCMH 5, GP Contractor  *it's actually a circular process around the patient, so it's definitely a more true team as opposed to just posting out the patient to see somebody and then you never hear anything back. There's more increased amounts of discussion about the patient, things like case conferencing. PCMH 5, GP Contractor  *Improving the patient-team partnership as well, that has been really important. So getting the patients more involved in their care team as well so that they build up a relationship with not just the doctors but the nurses and the dietician and the exercise physiologist as well. PCMH 5, GP Contractor  *It will become more like a teamwork approach with the patient problems and I think it might be very, very useful for the patients themselves ICP 4, Practice Principal  *it would be easier if we had these [team] allocations…Then the patient is properly looked after, things don’t get missed and slipped through the cracks. PCMH 6, GP Contractor  *we’ve got two pharmacists, one who speaks Tamil. So to talk to people in their own language to see about the medications they are taking and explain to them more clearly, so purposely get people who know the culture, the language to manage these patients. PCMH 2, Practice Principal B  *In a way we learn more about the patient through having them speak to different people, like when they’ve been seeing the same doctor for so long, sometimes things get forgotten or pushed under the rug, but when it is new eyes, it’s like okay, this is an issue, this is something that needs to be done and so that gets brought to the attention of the doctors, or to me who brings it to the attention of the doctors PCMH 8, Practice Nurse  *if a nurse reinforces the same thing and maybe a third person, allied health person reinforces the same thing, the chances are they’re more likely to accept or be willing to accept what you’re saying…I think the more people involved in care saying the same sort of thing, the better it would be. Non-PCMH 6, Practice Principal  *I'm quite happy for any other team member to review a patient and then advise me and of course we can then discuss about a much more comprehensive management plan for a lot of patients. So I think that we can actually provide a much more intensive service. PCMH 5, Practice Principal  *they're [patients] getting ability to see the other providers much more often. Other providers are able to help with reviewing care plans and management plans and things like that as well, so they're taking a more active role in the patient's health management condition. PCMH 5, GP Contractor  *The physician will have more time really to concentrate on establishing the framework where the patient can be monitored and managed instead of trying to do everything from the start to the end ICP 4, Practice Principal  *They [patients] don’t want the nurse involved. They just want to come in and see the doctor. So, that’s a slow process. Patients don’t change readily with what they’re used to, just seeing the doctor. “I don’t want to see the nurse, I just want to see you”. PCMH 6, GP Contractor  *it starts out in the waiting room, we’ll call their name and they’ll just be like “no, I’m here to see the doctor, I don’t want to see you”. That’s always a great feeling. PCMH 8, Practice Nurse  *We’ve had one or two people who have said, “No, I don’t care if it’s immunisation, I don’t want to see the nurse.” PCMH 6, Practice Principal  *it’s mainly the old people and they don’t like change at all PCMH 1, Practice Manager  *particularly the older patients have always had that care from the GP and it’s a transition for them to have a nurse do more or a medical assistant do more, plus trying to get them in…the attitude is often of, “I don’t need to see the nurse. Doctor so and so looks after that for me.” PCMH 4, Practice Manager |
| Practices | |
| **Staff satisfaction**   - *PCMH model of care helps improve job satisfaction* - *Staff satisfaction through greater service delivery* - *Professional and personal growth of staff* - *Staff have a sense of achievement* - *Working together as a team* - *Sharing the care frees up GPs time* - *Satisfaction through using skills* - *Delegation of care to the appropriate team member* - *Satisfaction through continuity of care* | *[PCMH model] it's a worthwhile approach for a few reasons and it kind of goes back to that quadruple aim that everyone talks about, that mainly it's going to improve your own job satisfaction. You've got to get up and go to work every day so if you're going to last for 30 years in general practice or 40 years you need to have good job satisfaction PCMH 5, GP Contractor  *this is the very first practice that actually I've worked in this situation of the PCMH…I agree with it. I like it. I came along and I enjoy working more than I used to… It's very rewarding because you get to see a lot more progress. PCMH 5, Dietician  *there's no question in my mind that there are rewards. There are very strong professional rewards and it allows you to evolve to what I would consider to be a higher level of service, of general practice service delivery. PCMH 5, Practice Principal  *the hard work needs to have a sense of achievement so at the end of every day, yeah, done this, done this, made a difference in people’s lives PCMH 4 Practice Principal  *It's improving their job satisfaction and involvement in the whole team because they actually become part of the team when they're working like that. PCMH 5, GP Contractor  *it’s good to share the patient care with others too so it frees some of our time, so someone else can look after the patients. ICP 5, Practice Principal  *it gets the nurse’s involved and the nurses feel really very happy about that because their skills are used. PCMH 2, Practice Principal B  *it would be such a better sense of job satisfaction, because all the things that can be done by someone else are being done by someone else, and the patient’s still coming back and getting good care PCMH 4, Practice Principal  *On the longer run, I think it will give greater satisfaction to the doctors – currently their frustration is about patient comes here, they see them, and then suddenly patient has been going and seeing somebody else also, getting some other advice, doing something else, and then comes back again after some time. So, that coordinated care, I think for the majority of the doctors eventually when they see it happening, it will give them that greater satisfaction. PCMH 7, Practice Manager |
| **Staff skills**   - *Sharing the care enables all staff skills to be utilised more effectively* - *Nursing skills are valued* - *Upskilling of staff* - *Multiskilling of staff* - *Career progression of staff* | *[team-based care] will allow our practice nurses to do some of those things and we’ll be able to utilise our allied health staff a lot more. Non-PCMH 7, Practice Principal  *so we promote them and say practice nurses this is what you do and you are a carer and you are a clinician and you help us. PCMH 2, Practice Principal B  *they're [front desk staff] very keen to be upskilled and to do different things. PCMH 5, GP Contractor  *what we had was a really interesting progression, because our receptionist was learning about cholesterol targets and blood pressure targets and how often all these different tests should be done, so it was a good learning curve for her PCMH 3, Practice Manager  *I'm a dietician. So as well as providing a service then I started upskilling as well. So I started doing spirometry, I started doing the care plan together with [name of Practice Principal], I started doing reviews of patients with diabetes and heart failure and respiratory disease. PCMH 5, Dietician  *one receptionist - I’m paying her to do a practice manager course. So hopefully, in 12 months she’ll become a practice manager and then she can start helping me with managing the practice. ICP 5, Practice Principal  *We’ve got one of the girls doing the medical assistant course so that means that she will then be qualified to help [name of nurse]. PCMH 1, Practice Manager  *We’ve got our senior receptionist going through the medical assistant course which has been fantastic and we also had one of our receptionists who became part of Team One. PCMH, 3 Practice Manager |
| **Use of care plans**   - *Care plans driving holistic, preventative care* - *Teamwork involved in carrying out care plans* - *Utilising computer software to improve care planning* - *Value of care plans in guiding care and not in financial remuneration* | *I made up a spreadsheet so that as we enrolled patients we could all have a meeting, discuss what the conditions were, what the targets for treatment were and also, how regularly some tests should be done. I designed the spreadsheets so that our receptionist could look and see what was about to fall due or if anything was overdue and manage that at reception with the patient and then the next step would be, does the medical assistant need to do anything with the patient? Or did the practice nurse need to work with the patient, because it exceeded the scope of the medical assistant. And then once the practice nurse has done everything she can do, then the patient gets to [Practice Principal] PCMH 3, Practice Manager  *we were discussing with two of my nurses and one of my other doctors and the practice manager how we are going to institute pre-planning, where I have just given permission for the nurses to go through my list of patients booked in for tomorrow, concentrate and be driven by the care-plan of those patients who, particularly with those ones that have got chronic disease and have a look at what can be done opportunistically in terms of preventative care, being evidenced-based preventive care, as per the guidelines and go for your life. PCMH 6, Practice Principal  *utilising the computer systems that we do have to improve our planned care…the medical software can be used to plan the care better for all the patients. PCMH 5, GP Contractor  *we have systems where we see a GP management plan that’s due six monthly, then it would be quarantined to book in for the chronic disease nurse. PCMH 3, Practice Principal  *everyone having diabetes, asthma, they were flagged that needs Asthma Cycle of Cares, care plans, spirometries. So when things are flagged as a reminder, it will actually appear on the patient appointment list and the nurse can see them and the doctor can see them. PCMH 8, Practice Principal  *hopefully it is a chronic home for like doing 721, 723 which is a care plan and I think it makes it like more productive, not just a $250 billing but it’s really like looking after the patient. Non-PCMH 3, Practice Manager |
| Health systems and costs | |
| **Integrated Care**   - *Integrating one model of care* - *Saving costs through one integrated system* - *Co-location of health care providers*      - *Taking pressure off hospitals with integrated model of care* - *Integrated specialist care for chronic disease patients* | *I think you need one, rather than having all these millions of silos operating separately, which is happening at the moment, you need to bring them altogether PCMH 7, GP contractor  *we have all the allied health professionals...I think with the Patient Centred Health Care Home they might integrate into the system in a more efficient way ICP 4, Practice Principal  *if we get a system that lets us see what happens to them [patients] when they’re not with us in terms of health sense, then I know we can save so much more money…we have to just really get one system or a system that will talk to other systems PCMH 4, Practice Principal  *The other benefit is having colleagues in house so that you can talk to them directly face-to-face which I find more useful, and you find it’s easier to contact them and get hold of them ICP 3, Practice Principal  *the population’s growing very fast in the Western area so we need to cater for that and we need to work smarter together, because if you work smarter in that group’s environment and you can register with the allied health service, the specialist working as a team connecting together and taking the load from the hospital as much as possible that will definitely have some positive impact on the patient. Non-PCMH 1, Practice Principal  *I think you should have western Sydney chronic disease home and provide a multi-disciplinary team of doctors and allied health professionals to look after chronic disease. Those people who are eligible should be followed by a multi-disciplinary team on a continuous basis. Non-PCMH 2, Practice Principal |
| **Reduced hospitalisations**   - *Reducing hospital admissions through continuity of care* - *Saving costs by reducing hospital admissions* - *Improving overall health system outcomes* | *continuity of care will mean less hospital presentations, better managed health PCMH 2, Practice Principal B  *The general practitioner will work more efficiently preventing the patient from re-admissions so I can see less hospitalisation, the patient is not presenting to the hospital for unnecessary things. Non-PCMH 1, Practice Principal  *the integrated care stuff does put a lot of focus on the patient and…gets them everything they need and just tries to keep them out of nursing homes and hospitals for as long as possible PCMH 8, Practice Nurse  *my absolutely conviction is that we already save so much money because we just don’t have patients go to hospital, it’s hardly ever, we take such good care of them PCMH 4, Practice Principal  *I will go so far as to say for the health system as a whole that if we can reduce hospitalisations you can improve overall outcomes. PCMH 5, Practice Principal |
| **Reduced duplication**   - *Saving costs by reducing duplication of tests* - *Need for restricting number of doctors a patient can see* - *Saving costs by registering patients* | *if I knew that they’d done that test I wouldn’t have to repeat it…and save a bucket load of money in tests that don’t need to be repeated PCMH 4, Practice Principal  *the government wants to make a system that’s efficient, not duplicating and that is promoting consistent health care. PCMH 7, GP Contractor  *patients can go to three different doctors on the same day and all three do not talk to each other and they’ll be given three different types of treatment. PCMH 2, Practice Principal B  *[Patient registration] would make a lot of difference. It will save the government a lot of money, you don’t have all of this shopping around buying drugs here and there…We have so much doubling, we have so much programs. At the end of the day we just need a one stop shop…ultimately the health dollar will be much less because the patient will be restricted to three or four doctors. Non-PCMH 1, Practice Principal |
